# The microscopic relationships between triangular arbitrage and cross-currency correlations in a simple agent based model of foreign exchange markets

**DOI:** 10.1371/journal.pone.0234709

**Published:** 2020-06-24

**Authors:** Alberto Ciacci, Takumi Sueshige, Hideki Takayasu, Kim Christensen, Misako Takayasu

**Affiliations:** 1 Blackett Laboratory, Imperial College London, London, England, United Kingdom; 2 Center for Complexity Science, Imperial College London, London, England, United Kingdom; 3 Department of Mathematical and Computing Science, School of Computing, Tokyo Institute of Technology, Nagatsuta-cho, Midori-ku, Yokohama, Japan; 4 Institute of Innovative Research, Tokyo Institute of Technology, Nagatsuta-cho, Yokohama, Japan; 5 Sony Computer Science Laboratories, Higashigotanda, Shinagawa-ku, Tokyo, Japan; University of Almeria, SPAIN

## Abstract

Foreign exchange rates movements exhibit significant cross-correlations even on very short time-scales. The effect of these statistical relationships become evident during extreme market events, such as flash crashes. Although a deep understanding of cross-currency correlations would be clearly beneficial for conceiving more stable and safer foreign exchange markets, the microscopic origins of these interdependencies have not been extensively investigated. This paper introduces an agent-based model which describes the emergence of cross-currency correlations from the interactions between market makers and an arbitrager. The model qualitatively replicates the time-scale vs. cross-correlation diagrams observed in real trading data, suggesting that triangular arbitrage plays a primary role in the entanglement of the dynamics of different foreign exchange rates. Furthermore, the model shows how the features of the cross-correlation function between two foreign exchange rates, such as its sign and value, emerge from the interplay between triangular arbitrage and trend-following strategies. In particular, the interaction of these trading strategies favors certain combinations of price trend signs across markets, thus altering the probability of observing two foreign exchange rates drifting in the same or opposite direction. Ultimately, this entangles the dynamics of foreign exchange rate pairs, leading to cross-correlation functions that resemble those observed in real trading data.

## 1 Introduction

Various non-trivial statistical regularities, known as *stylized facts* [1], have been documented in trading data from markets of different asset classes [2]. The available literature examined the heavy-tailed distribution of price changes [3–6], the long memory in the absolute mid-price changes (volatility clustering) [4, 6–10], the long memory in the direction of the order flow [10–13] and the absence of significant autocorrelation in mid-price returns time series, with the exclusion of negative, weak but still significant autocorrelation observed on extremely short time-scales [6, 9, 14–16]. Different research communities (e.g., physics, economics, information theory) took up the open-ended challenge of devising models that could reproduce these regularities and provide insights on their origins [2, 17, 18]. Economists have traditionally dealt with optimal decision-making problems in which perfectly rational agents implement trading strategies to maximize their individual utility [2, 17, 18]. Previous studies have looked at cut-off decisions [19–21], asymmetric information and fundamental prices [22–26] and price impact of trades [27–30]. In the last thirty years the orthodox assumptions of full rationality and perfect markets have been increasingly disputed by emerging disciplines, such as behavioral economics, statistics and artificial intelligence [17]. The physics community have also entered this *quest for simple models of non-rational choice* [17] by taking viewpoints and approaches, such as zero-intelligence and agent-based models, that often stray from those that are common among economists. Agent-based models (ABMs henceforth) rely on simulations of interactions between agents whose actions are driven by idealized human behaviors [17]. A seminal attempt to describe agents interactions through ABM is the *Santa Fe Stock Market* [31], which neglects the perfect rationality assumption by taking an artificial intelligence approach [17]. The model successfully replicates various stylized facts of financial markets (e.g., heavy-tailed distribution of returns and volatility clustering), hinting that the lack of full rationality has a primary role in the emergence of these statistical regularities [17]. Following [31], several ABMs [32–44] have further examined the relationships between the microscopic interactions between agents and the macroscopic behavior of financial markets.

This study introduces a new ABM of the foreign exchange (FX henceforth) market. The FX market is characterized by singular institutional features, such as the absence of a central exchange, exceptionally large traded volumes and a declining, yet significant dealer-centric nature [45]. Electronic trading has rapidly emerged as a key channel through which investors can access liquidity in the FX market [45, 46]. For instance, more than 70% of the volume in the FX Spot market is exchanged electronically [46]. A peculiar stylized fact of the FX market is the significant correlation among movements of different currency prices. These interdependencies are time-scale dependent [47, 48], their strength evolves in time and become extremely evident in the occurrence of extreme price swings, known as flash crashes. In these events, various foreign exchange rates related to a certain currency abruptly appreciate or depreciate, affecting the trading activity of several FX markets. A recent example is the large and rapid appreciation of the Japanese Yen against multiple currencies on January 2^nd^ 2019. The largest intraday price changes peaked +11% against Australian Dollar, +8% against Turkish Lira and +4% against US Dollar [49]. The relationship between triangular arbitrage [50–53] and cross-currency correlations remains unclear. Mizuno *et al*. [47] observed that the cross-correlation between real and implied prices of Japanese Yen is significantly below the unit on very short time-scales, conjecturing that this counter-intuitive property highlights how the same currency could be purchased and sold at different prices by implementing a triangular arbitrage strategy. Aiba and Hatano [37] proposed an ABM relying on the intriguing idea that triangular arbitrage influences the price dynamics in different currency markets. However, this study fails to explain whether and how reactions to triangular arbitrage opportunities lead to the characteristic shape of the time-scale vs. cross-correlation diagrams observed in real trading data [47, 48].

Building on these observations, the present study aims to obtain further insights on the microscopic origins of the correlations among currency pairs by introducing an ABM model in which two species (i.e., market makers and the arbitrager) interact across three inter-dealer markets where trading is organized in limit order books. The model qualitatively replicates the characteristic shape of the cross-correlation functions between currency pairs observed in real trading data. This suggests that triangular arbitrage is a pivotal microscopic mechanism behind the formation of cross-currency interdependencies. Furthermore, the model elucidates how the features of these statistical relationships, such as the sign and value of the time-scale vs. cross-correlation diagram, stem from the interplay between trend-following and triangular arbitrage strategies.

This paper is organized as follows. Section 2 outlines the basic concepts, discusses the employed dataset and provides a detailed description of the proposed model. Section 3 examines the behavior of the model in order to collect insights on the microscopic origins of cross-currency interdependencies. Section 4 concludes and provides an outlook on the research paths that could be developed from the outcomes of this study. Technical details, further empirical analyses and an extended version of the model are presented in the supporting information sections.

## 2 Methods

### 2.1 Concepts

#### 2.1.1 Limit order books

**Fig 1:**
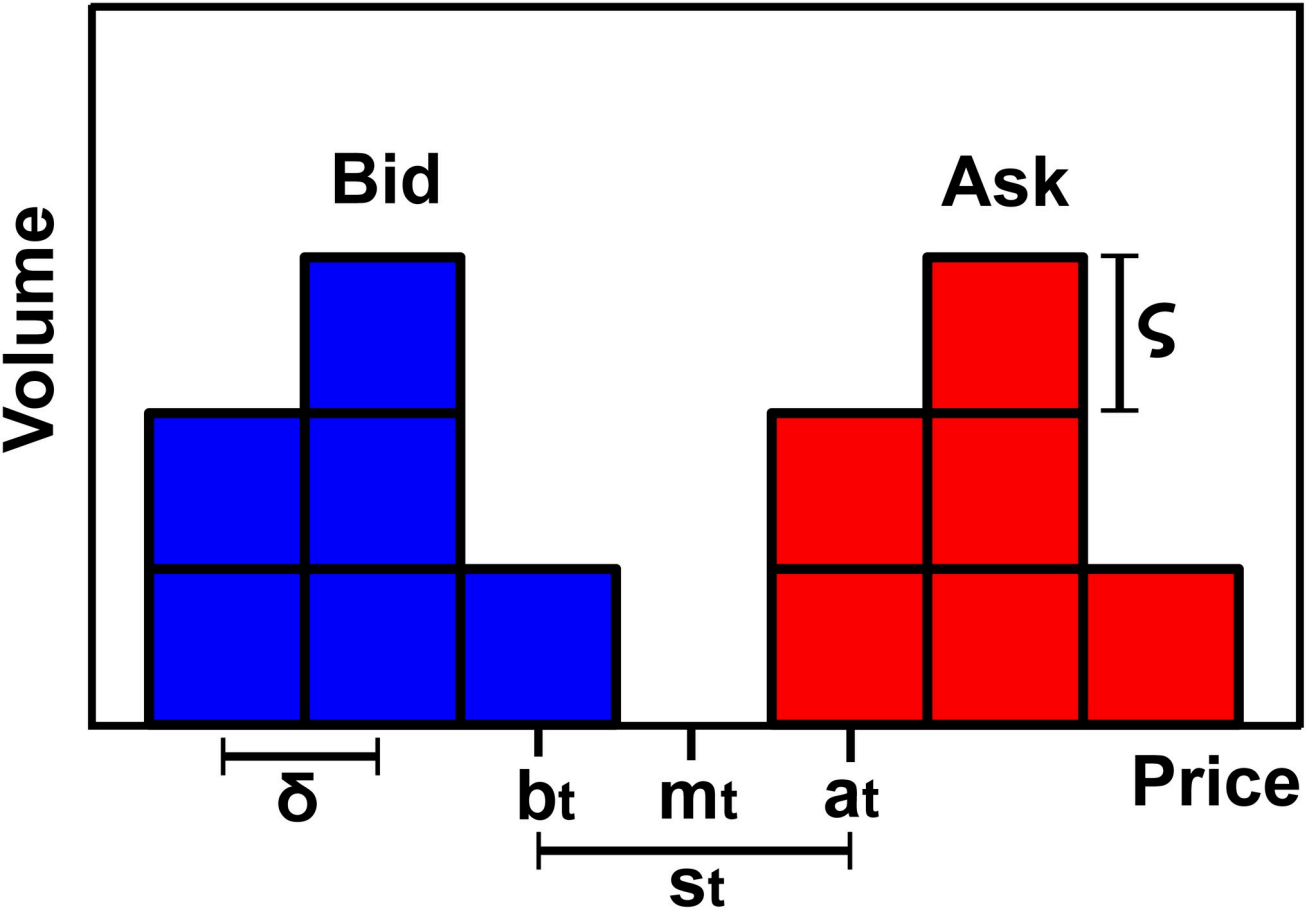
Schematic of a LOB and related terminology. At any time *t* the *bid price* *b*_*t*_ is the highest limit price among all the buy limit orders (blue) while the *ask price* *a*_*t*_ is the lowest limit price among all the sell limit orders (red). The bid and ask prices are the *best quotes* of the LOB. The mid point between the best quotes *m*_*t*_ = (*a*_*t*_ + *b*_*t*_)/2 is the *mid price*. The distance between the best quotes *s*_*t*_ = *a*_*t*_ − *b*_*t*_ is the *bid-ask spread*. The volume specified in a limit order must be a multiple of the *lot size* *ς*, which is the minimum exchangeable quantity (in units of the traded asset). The price specified in a limit order must be a multiple of the *tick size* *δ*, which is the minimum price variation imposed by the LOB. The lot size *ς* and the tick size *δ* are known as *resolution parameters* of the LOB [2]. Orders are allocated in the LOB depending on their distance (in multiples of *δ*) from the current best quote. For instance, a buy limit order with price *b*_*t*_ − *nδ* occupies the (*n* + 1)-th level of the bid side.

Electronic trading takes place in an online platform where traders submit buy and sell orders for a certain assets through an online computer program. Unmatched orders *await* for execution in electronic records known as limit order books (LOBs henceforth), see Fig 1. By submitting an order, traders pledge to sell (buy) up to a certain quantity of a given asset for a price that is greater (less) than or equal to its limit price [2, 54]. The submission activates a trade-matching algorithm which determines whether the order can be immediately matched against earlier orders that are still queued in the LOB [54]. A matching occurs anytime a buy (sell) order includes a price that is greater (less) than or equal to the one included in a sell (buy) order. When this occurs, the owners of the matched orders engage in a transaction. Orders that are completely matched upon entering into the system are called *market orders*. Conversely, orders that are partially matched or completely unmatched upon entering into the system (i.e., *limit orders*) are queued in the LOB until they are completely matched by forthcoming orders or deleted by their owners [54].

The limit order with the best price (i.e., the highest bid or the lowest ask quote) is always the first to be matched against a forthcoming order. The adoption of a minimum price increment *δ* forces the price to move in a discrete grid, hence the same price can be occupied by multiple limit orders at the same time. As a result, exchanges adopt an additional rule to prioritize the execution of orders bearing the same price. A very common scheme is the *price-time* priority rule which uses the submission time to set the priority among limit orders occupying the same price level, i.e., the order that entered the LOB earlier is executed first [54].

#### 2.1.2 Triangular arbitrage

**Fig 2:**
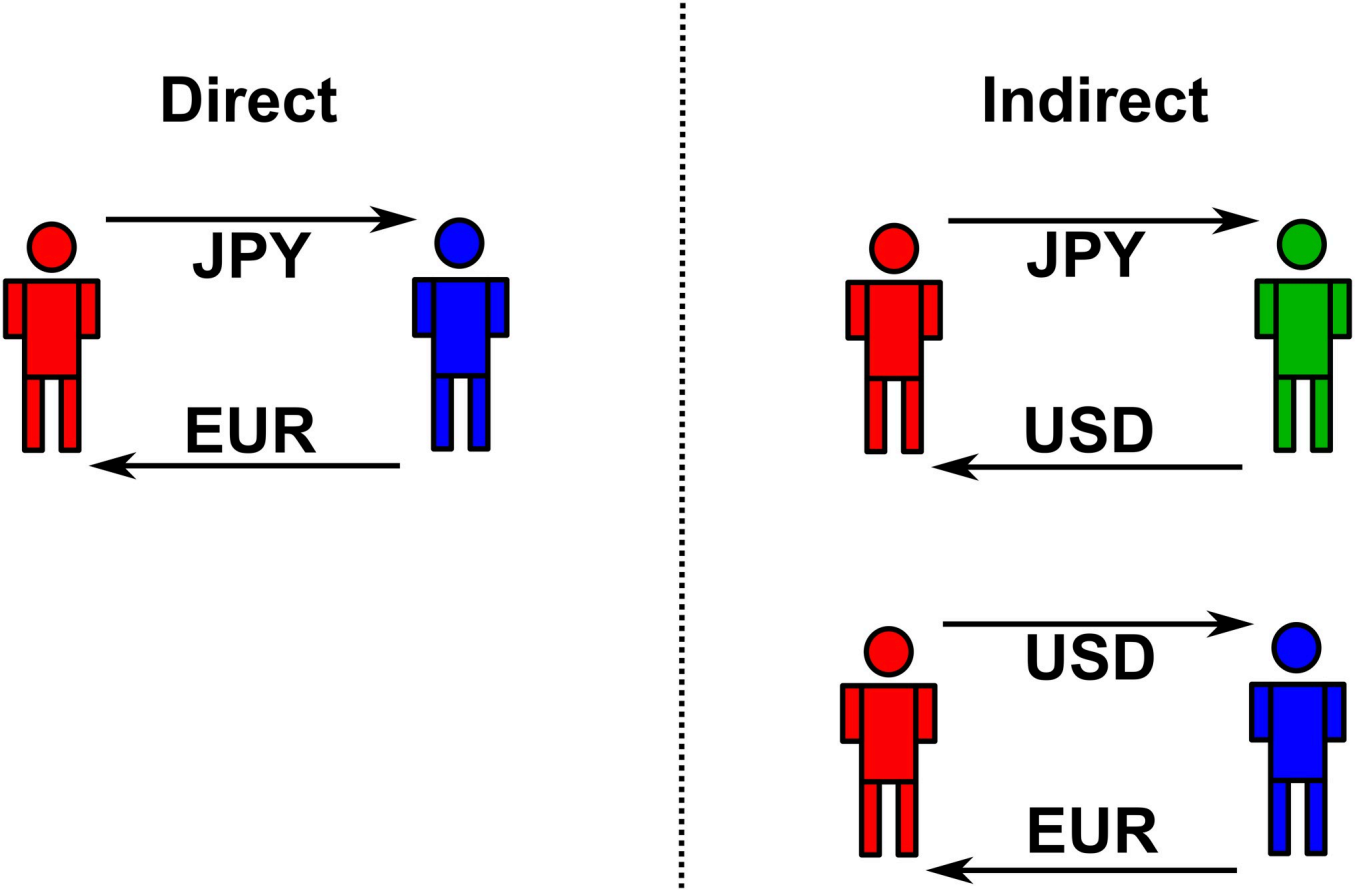
Two ways of obtaining one unit of EUR. Direct transaction (left panel): agent #1 (red) obtains EUR from agent #2 (blue) in exchange for JPY. Indirect transaction (right panel): agent #1 purchases USD from agent #3 (green) in exchange for JPY. Then, she obtains EUR from agent #2 (blue) in exchange for USD.

In the FX market, the price of a currency is always expressed in units of another currency and it is commonly known as foreign exchange rate (FX rate henceforth). For instance, the price of one Euro (EUR henceforth) in Japanese Yen (JPY henceforth) is denoted by EUR/JPY. The same FX rate can be obtained from the product of two other FX rates, e.g., EUR/JPY = USD/JPY × EUR/USD, where USD indicates US Dollars. In the former case EUR is purchased directly while in the latter case EUR is purchased indirectly through a third currency (i.e., USD), see Fig 2.

At any time *t* we expect the following equality to hold

$$\begin{array}{c} \underset{\text{FX}\;\text{rate}}{\underset{︸}{\text{EUR}/{\text{JPY}}_{t}}}=\underset{\text{implied}\;\text{FX}\;\text{cross}\;\text{rate}}{\underset{︸}{\left(\text{USD}/{\text{JPY}}_{t}\right)\times \left(\text{EUR}/{\text{USD}}_{t}\right)}}, \end{array}$$ (1)

that is, the costs of a direct and indirect purchase of the same amount of a given currency must be the same. Clearly, Eq (1) can be generalized to any currency triplet.

However, several datasets [50, 51, 53, 55] reveal narrow time windows in which Eq (1) does not hold. In this scenario, traders might try to exploit one of the following misprices

$$\begin{array}{c} \text{EUR}/{\text{JPY}}_{t}<\left(\text{USD}/{\text{JPY}}_{t}\right)\times \left(\text{EUR}/{\text{USD}}_{t}\right), \end{array}$$ (2a)

$$\begin{array}{c} \text{EUR}/{\text{JPY}}_{t}>\left(\text{USD}/{\text{JPY}}_{t}\right)\times \left(\text{EUR}/{\text{USD}}_{t}\right), \end{array}$$ (2b)

by implementing a triangular arbitrage strategy. For instance, Eq (2b) suggests that a trader holding JPY could gain a risk-free profit by buying EUR indirectly (JPY → USD → EUR) and selling EUR directly (EUR → JPY).

**Fig 3:**
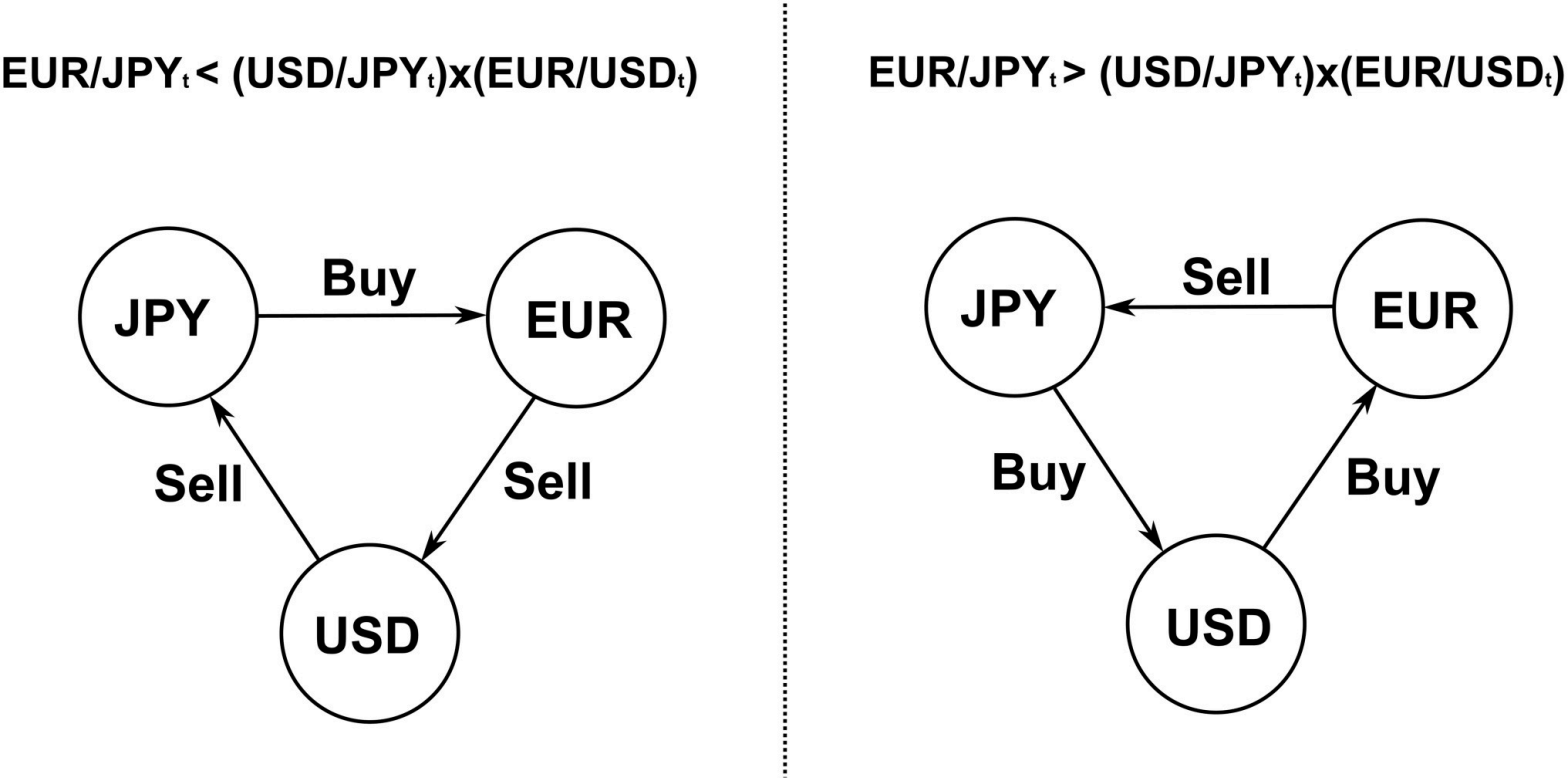
Profitable misprices and associated triangular arbitrage strategies. Left panel: an agent buys EUR for JPY and sells EUR for JPY through USD. Right panel: an agent sells EUR for JPY and buys EUR for JPY through USD.

Assuming that the arbitrager completes each transaction at the best quotes (i.e., sell at the best bid and buy at the best ask) available in the EUR/JPY, USD/JPY and EUR/USD LOBs, any strategy presented in Fig 3 is effectively profitable if the following condition (i.e., Eq (3a) for left panel strategy or Eq (3b) for right panel strategy) is satisfied

$$\begin{array}{c} a_{\text{EUR}/\text{JPY}}\left(t\right)<b_{\text{USD}/\text{JPY}}\left(t\right)\times b_{\text{EUR}/\text{USD}}\left(t\right), \end{array}$$ (3a)

$$\begin{array}{c} b_{\text{EUR}/\text{JPY}}\left(t\right)>a_{\text{USD}/\text{JPY}}\left(t\right)\times a_{\text{EUR}/\text{USD}}\left(t\right), \end{array}$$ (3b)

where *b*_*x*/*y*_(*t*) and *a*_*x*/*y*_(*t*) are the best bid and ask quotes available at time *t* in the *x*/*y* market respectively.

Following [37, 50, 51], the presence of triangular arbitrage opportunities is detected whenever one of the following processes

$$\begin{array}{c} \mu^{I}\left(t\right)=\frac{b_{\text{USD}/\text{JPY}}\left(t\right)\times b_{\text{EUR}/\text{USD}}\left(t\right)}{a_{\text{EUR}/\text{JPY}}\left(t\right)}, \end{array}$$ (4a)

$$\begin{array}{c} \mu^{II}\left(t\right)=\frac{b_{\text{EUR}/\text{JPY}}\left(t\right)}{a_{\text{USD}/\text{JPY}}\left(t\right)\times a_{\text{EUR}/\text{USD}}\left(t\right)}, \end{array}$$ (4b)

exceeds the unit. The terms *b*_*x*/*y*_(*t*) and *a*_*x*/*y*_(*t*) retain the same meaning as in Eq (3).

### 2.2 The EBS dataset

This study employs highly granular LOB data provided by Electronic Broking Services (EBS henceforth). EBS is an important inter-dealer electronic platform for FX spot trading [46]. Trading is organized in LOBs and estimates suggest that approximately 70% of the orders are posted by algorithms [46]. The empirical analysis presented in this work covers three major FX rates, USD/JPY, EUR/USD and EUR/JPY, across four years of trading activity (2011-2014). The EBS dataset provides a 24-7 coverage of the trading activity (from 00:00:00.000 GMT Monday to 23:59:59.999 GMT Sunday included), thus offering a complete and uninterrupted record of the flow of submissions, executions and deletions occurring in the first ten price levels of the bid and ask sides of the LOB, see Table 1 for further details.

**Table 1:** EBS dataset structure.

Date	Timestamp	Market	Event	Direction	Depth	Price	Volume
2011-05-10	09.00.00.000	USD/JPY	Deal	Buy	1st	100.000	1
⋮	⋮	⋮	⋮	⋮	⋮	⋮	⋮
2011-10-21	21.00.00.000	EUR/USD	Quote	Ask	3rd	0.8000	5

Each record (i.e., row) corresponds to a specific market event. Records are reported in chronological order (top to bottom) and include the following details: i) date (yyyy-mm-dd), ii) timestamp (GMT), iii) the market in which the event took place, iv) event type (submission (Quote) or execution (Deal) of visible or hidden limit orders), v) direction of limit orders (Buy/Sell for deals and Bid/Ask for quotes), vi) depth (number of occupied levels) between the specified price and the best price, vii) price and viii) units specified in the limit order.

The shortest time window between consecutive records is 100 millisecond (ms). Events occurring within 100 ms are aggregated and recorded at the nearest available timestamp. The tick size has changed two times within the considered four years window, see [56] and S1 Table in S1 File for further details.

The EBS dataset, in virtue of its features, is a reliable source of granular market data. First, EBS directly collects data from its own trading platform. This prevents the common issues associated to the presence of third parties during the recording process, such as interpolations of missing data and input errors (e.g., incorrect timestamps or order types). Second, the EBS dataset offers a continuous record of LOB events across a wide spectrum of currencies, thus becoming a natural choice for cross-sectional studies (e.g., triangular arbitrage or correlation networks). Third, in spite of the increasing competition, the EBS platform has remained a key channel for accessing FX markets for more than two decades by connecting traders across more than 50 countries [57, 58]. The enduring relevance of this platform has been guaranteed by the fairness and the competitiveness of the quoted prices.

### 2.3 The Arbitrager Model

**Fig 4:**
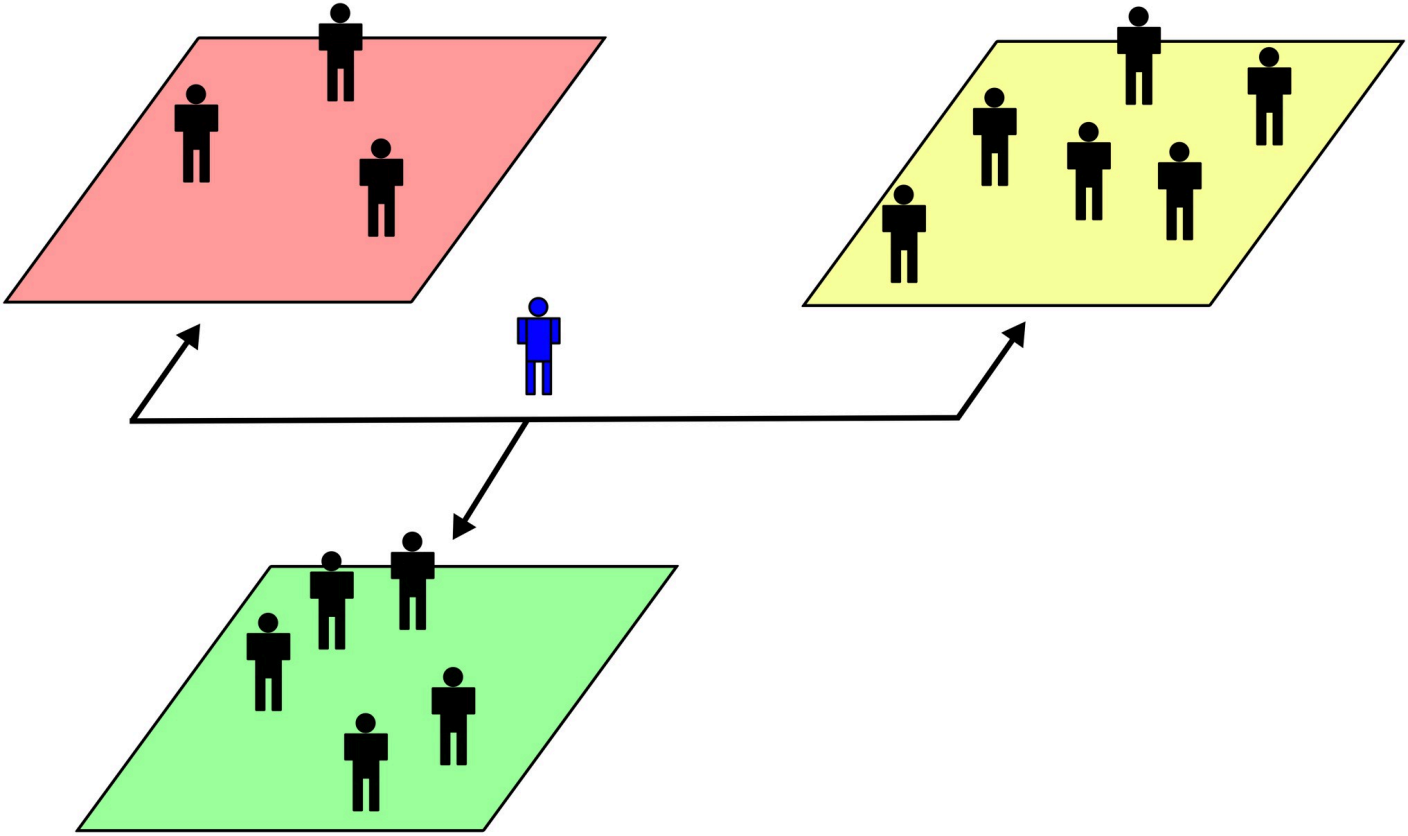
Schematic of the Arbitrager Model ecology. The ecology comprises three independent FX markets represented by the red, yellow and green areas. For simplicity, each market allows to exchange a major FX rate: USD/JPY, EUR/USD and EUR/JPY. Trading is organized in continuous price grid LOBs as in [42], see S3.1 Section. Market makers (black agents) maintain bid and ask quotes by adopting trend-based strategies. Transactions occur when the best bid matches or exceeds the best ask. Market makers engaging in a trade close the deal at the mid point between the two matching prices (i.e., transaction price), see S4 Fig for details. Finally, an arbitrager (blue agent) exclusively submits market orders across the three markets (black arrows) to exploit triangular arbitrage opportunities emerging now and then, see S5 Fig.

To meet the goals of this study, a model (Arbitrager Model henceforth) of three co-existing inter-dealer FX markets is introduced. The scope of this framework is to mimic the interactions between different trading strategies across multiple FX markets and capture the mechanisms through which these interactions shape the documented cross-correlation among FX rate fluctuations [47, 48]. In the Arbitrager Model, each market hosts a fixed number of agents who interact by exchanging a given FX rate. Trading is organized in simplified LOBs where prices move in a continuous grid. Agents provide liquidity to the market by adjusting limit orders through which they quote a bid and an ask price, thus acting as market makers. To set these prices, market makers adopt simple trend-based strategies. Furthermore, market makers cannot interact across markets, that is, they can only trade in the market they have been assigned to. Finally, echoing [37], the ecology hosts a special agent (i.e., the arbitrager) that is allowed to submit market orders in any market to exploit triangular arbitrage opportunities, see Fig 4.

#### 2.3.1 Market makers

The *i*-th market maker operating in the *ℓ*-th market actively manages a bid quote *b*_*i*,*ℓ*_(*t*) and an ask quote *a*_*i*,*ℓ*_(*t*) separated by a constant spread *L*_*ℓ*_ = *a*_*i*,*ℓ*_(*t*) − *b*_*i*,*ℓ*_(*t*). To do so, the *i*-th market maker updates its dealing price *z*_*i*,*ℓ*_(*t*), which is the mid point between the two quotes (i.e., *z*_*i*,*ℓ*_(*t*) = *a*_*i*,*ℓ*_(*t*) − *L*_*ℓ*_/2 = *b*_*i*,*ℓ*_(*t*) + *L*_*ℓ*_/2), by adopting a trend-based strategy

$$\begin{array}{c} \frac{dz_{i,\ell }\left(t\right)}{dt}=c_{\ell }\phi_{n,\ell }\left(t\right)+\sigma_{\ell }\epsilon_{i,\ell }\left(t\right),\;\;\;i=1,\cdots ,N_{\ell } \end{array}$$ (5)

where *N*_ℓ_ is the number of market makers participating the *ℓ*-th market, *σ*_*ℓ*_ > 0, and *ϵ*_*i*,*ℓ*_(*t*) is a Gaussian white noise. The term

$$\begin{array}{c} \phi_{n,\ell }\left(t\right)=\frac{\sum_{k=0}^{n-1}(p_{\ell }\left(g_{t,\ell }-k\right)-p_{\ell }\left(g_{t,\ell }-k-1\right))e^{-\frac{k}{\xi }}}{\sum_{k=0}^{n-1}e^{-\frac{k}{\xi }}},\;\;\;\ell =1,\ldots ,d \end{array}$$ (6)

is the weighted average of the last *n* < *g*_*t*,*ℓ*_ changes in the transaction price *p*_*ℓ*_ in the *ℓ*-th market and *g*_*t*,*ℓ*_ is the number of transactions occurred in [0, *t*[in the *ℓ*-th market. In this average, the weight $e^{-\frac{k}{\xi }}$ decays exponentially fast with characteristic scale *ξ* > 0. The real-valued parameter *c*_*ℓ*_ controls how the current price trend *ϕ*_*n*,*ℓ*_(*t*) influences market makers’ strategies. For instance, *c*_*ℓ*_ > 0 (*c*_*ℓ*_ < 0) indicates that market makers operating in the *ℓ*-th market tend to adjust their dealing prices *z*(*t*) in the same (opposite) direction of the sign of the price trend *ϕ*_*n*,*ℓ*_(*t*).

Transactions occur when the *i*-th market maker is willing to buy at a price that matches or exceeds the ask price of the *j*-th market maker (i.e., *b*_*i*,*ℓ*_ ≥ *a*_*j*,*ℓ*_). Trades are settled at the transaction price *p*(*g*_*t*,*ℓ*_) = (*a*_*j*,*ℓ*_(*t*) + *b*_*i*,*ℓ*_(*t*))/2 and only the market makers who have just engaged in a trade adjust their dealing prices *z*(*t* + *dt*) to the latest transaction price *p*(*g*_*t*,*ℓ*_), see S3 and S4 Figs.

#### 2.3.2 The arbitrager

The arbitrager is a liquidity taker (i.e., she does not provide bid and ask quotes like market makers) that can only submit market orders in each market to exploit an existing triangular arbitrage opportunity. Assuming that agents exchange EUR/JPY, EUR/USD and USD/JPY, the arbitrager monitors the triangular arbitrage processes presented in Eqs (4a) and (4b). As soon as one of these processes exceeds the unit, the arbitrager submits market orders to exploit the current opportunity (predatory market orders henceforth). Contrary to limit orders, market orders trigger an immediate transaction between the arbitrager and the market maker providing the best quote on the opposite side of the LOB. This implies that transactions involving the arbitrager are always settled at the bid or ask quote offered by the matched market maker, which are by the definition the current best bid or ask quote of the LOB. Following the post-transaction update rule, the matched market maker adjust its dealing price to its own matched bid or ask quote, that is, *z*_*i*,*ℓ*_(*t* + *dt*)→*a*_*i*,*ℓ*_(*t*) in case of a buy predatory market order or *z*_*i*,*ℓ*_(*t* + *dt*)→*b*_*i*,*ℓ*_(*t*) in case of a sell predatory market order, see S5 Fig.

#### 2.3.3 The Arbitrager Model in context

The Arbitrager Model builds on various existing studies. The structure of each market mimics, with few exceptions, the one introduced in the Dealer Model [42], where a number of autonomous market makers interact in a continuous price-grid LOB by managing limit orders. In the Arbitrager Model, the strategic behavior of market makers is driven by a simple process, see Eq (5), that is reminiscent of those proposed in the Dealer Model [42] and, more recently, in the HFT Model [59]. Finally, the idea of an arbitrager acting as a *connection* between otherwise independent markets was introduced in the Aiba and Hatano Model [37]. In particular, the authors advanced the intriguing comparison between an ecology comprising multiple markets, such as the Arbitrager Model, and a spring-mass system in which the dynamics of three random walkers (i.e., the markets) are constrained by a restoring force (i.e., the arbitrager) acting on the center of gravity of the system.

The Arbitrager Model presents the main features of the ABM approach [60]. First, it is composed by several actors (i.e., agents) who autonomously evaluate the current state of the system before taking a certain decision, such as re-adjusting their limit orders. Second, the decision making processes, the available trading strategies and the rules governing the interactions among agents retain a remarkable simplicity. This reduces the computational effort required to build and simulate the dynamics of the model and facilitates the understanding and interpretation of its outcomes. Third, the Arbitrager Model does not achieve its goal by directly modelling cross-currency correlations. Instead, this statistical regularity of FX markets is conceived as a macroscopic phenomenon which emerges from the iteration of simple, antagonistic interactions occurring on a more microscopic level.

## 3 Results

### 3.1 Cross-correlation functions

Echoing previous empirical studies [47, 48], the cross-correlation function between fluctuations of two foreign exchange rates is

$$\begin{array}{c} \rho_{i,j}\left(\omega \right)=\frac{\left\langle \Delta m_{i}\left(t\right)\Delta m_{j}\left(t\right)\right\rangle -\left\langle \Delta m_{i}\left(t\right)\right\rangle \left\langle \Delta m_{j}\left(t\right)\right\rangle }{\sigma_{\Delta m_{i}}\sigma_{\Delta m_{j}}}, \end{array}$$ (7a)

$$\begin{array}{c} \sigma_{\Delta m_{\ell }}^{2}=\left\langle \Delta m_{\ell }{\left(t\right)}^{2}\right\rangle -{\left\langle \Delta m_{\ell }\left(t\right)\right\rangle }^{2}, \end{array}$$ (7b)

where the time-scale *ω* is the interval (i.e., in seconds) between two consecutive observations of the *ℓ*-th mid price *m*_*ℓ*_ time series, Δ*m*_*ℓ*_(*t*)≡*m*_*ℓ*_(*t*) − *m*_*ℓ*_(*t* − *ω*) is the linear change between consecutive observations, $\sigma_{\Delta m_{\ell }}$ is the standard deviation of Δ*m*_*ℓ*_(*t*) and 〈〉 denotes average values.

**Fig 5:**
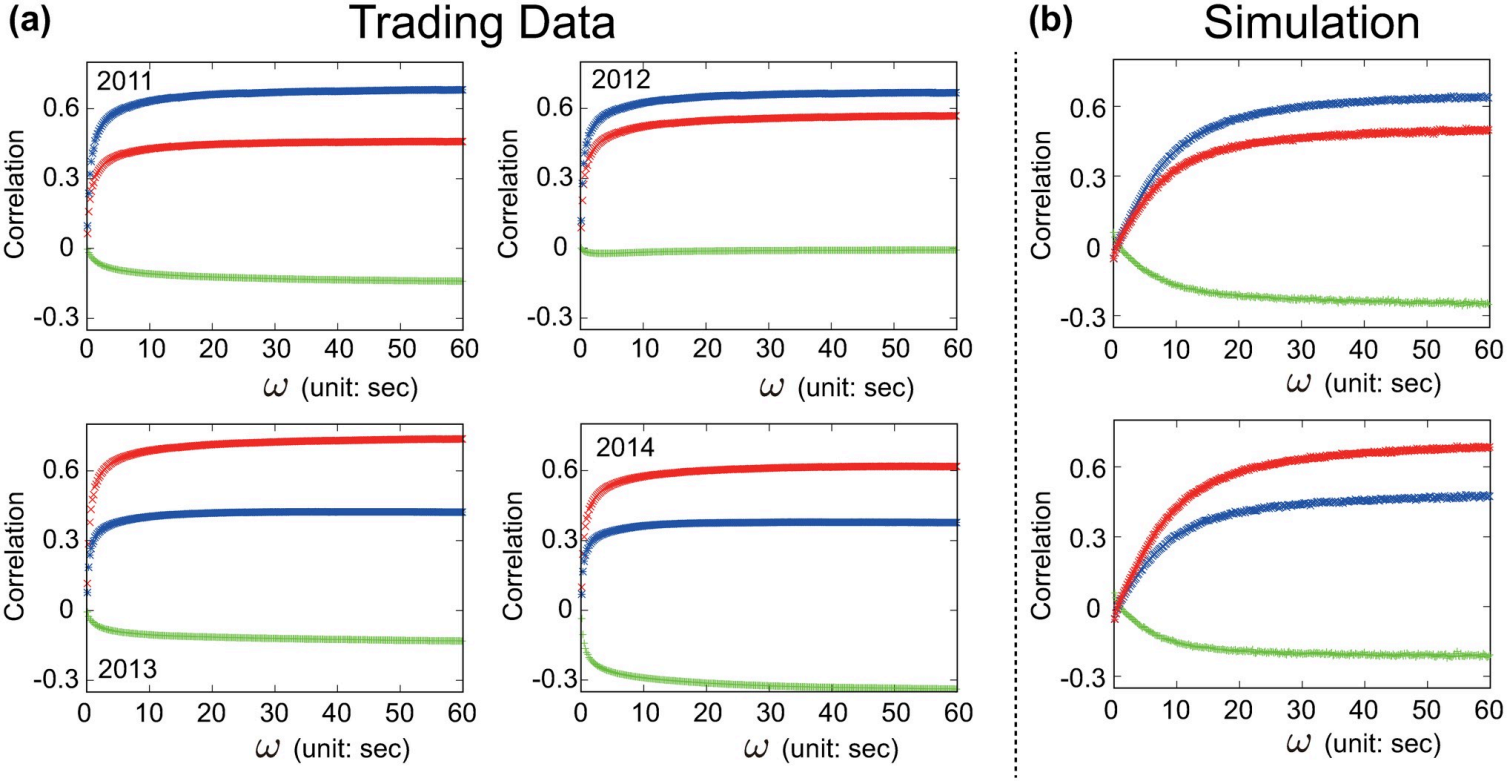
Trading data vs. model based cross-correlation functions. Cross-correlation function *ρ*_*i*,*j*_(*ω*) for ΔUSD/JPY vs. ΔEUR/USD (green), ΔEUR/USD vs. ΔEUR/JPY (blue) and ΔUSD/JPY vs. ΔEUR/JPY (red) as a function of the time-scale *ω* of the underlying time series. (a) Real market data (EBS) across four distinct years (2011-2014). (b) Arbitrager Model simulations. The number of participating market makers (*N*_EUR/USD_, *N*_USD/JPY_, *N*_EUR/JPY_) are (35, 45, 25) in the first experiment, see (b) top panel, and (50, 35, 25) in the second experiment, see (b) bottom panel. The trend-following strength parameters are (*c*_EUR/USD_, *c*_USD/JPY_, *c*_EUR/JPY_) = (0.8, 0.8, 0.8) in both experiments. The length of each simulation is 5 × 10^6^ time steps. The price trends *ϕ*_*n*,*ℓ*_ are calculated over the most recent *n* = 15 changes in the transaction price *p* and the scaling constant is set to *ξ* = 5, see Eq (6). Details on the initialization of the model and the conversion between simulation time (i.e., time steps) and real time (i.e., sec) are provided in S3.2 Section.

In real trading data, the value of the cross-correlation function *ρ*_*i*,*j*_(*ω*) varies with *ω* on very short time-scales (*ω* < 1 sec). This time-scale dependency starts to weaken after *ω* ≈ 1 sec and vanishes beyond *ω* ≈ 10 sec, see Fig 5(a). The characteristic shape of *ρ*_*i*,*j*_(*ω*) displayed in Fig 5(a) is compatible with the one found by Mizuno *et al*. [47]. However, the trading data-based cross-correlation functions presented in this study stabilize on much shorter time-scales. Considering that [47] employed trading data collected in 1999, a period where lower levels of automation imposed a slower trading pace, it is plausible to hypothesize that the time-scale *ω* beyond which *ρ*_*i*,*j*_(*ω*) stabilizes reflects the speed at which markets react to a given event. Furthermore, *ρ*_*i*,*j*_(*ω*) stabilizes around different levels over the four trading years covered in the present analysis. For instance, the cross-correlation between ΔUSD/JPY and ΔEUR/JPY, see Fig 5(a), stabilizes around 0.6 in 2011-2012 and 0.3 in 2013-2014. This variability might be related to the different tick sizes adopted by EBS during the four years covered in this empirical analysis, see [56] and S1 Table in S1 File. Detailed investigations on how changes in the design of FX LOBs (e.g., tick size) and the increasing sophistication of market participants (e.g., high frequency traders) affect the characteristic shape of *ρ*_*i*,*j*_(*ω*) are outside the scope of this paper, however, such studies will be a very much welcomed addition to the current literature.

The Arbitrager Model satisfactorily replicates the characteristic shape of *ρ*_*i*,*j*_(*ω*), suggesting that triangular arbitrage plays a primary role in the entanglement of the dynamics of currency pairs in real FX markets. However, two quantitative differences between the model-based and data-based characteristic shape of *ρ*_*i*,*j*_(*ω*) emerge in Fig 5. First, *ρ*_*i*,*j*_(*ω*) flattens after *ω* ≈ 30 sec in the model, see Fig 5(b), and *ω* ≈ 10 sec in real trading data, see Fig 5(a). Second, in extremely short time-scales (*ω* → 0 sec) the model-based *ρ*_*i*,*j*_(*ω*) does not converge to zero as in real trading data, see Fig 5(b), but to nearby values. These discrepancies might be rooted in the extreme simplicity of the Arbitrager Model which neglects various practices of real FX markets that contribute, to different degrees, to the shape and features of *ρ*_*i*,*j*_(*ω*) revealed in real trading data. To support this hypothesis, an extended version of the Arbitrager Model which includes additional features of real FX markets is presented and examined in S3.3 Section. This more complex version of the model overcomes the main differences between the curves displayed in Fig 5(a) and 5(b), reproducing cross-correlation functions *ρ*_*i*,*j*_(*ω*) that approach zero when *ω* → 0 sec and stabilize on shorter time-scales than those emerged in the baseline model.

### 3.2 The interplay between triangular arbitrage and trend-following strategies intertwines FX rates dynamics

The Arbitrager Model, reproducing the characteristic shape of *ρ*_*i*,*j*_(*ω*), suggests that triangular arbitrage plays a primary role in the formation of the cross-correlations among currencies. However, it is not clear how the features of *ρ*_*i*,*j*_(*ω*), such as its sign and values, stem from the interplay between the different types of strategies adopted by agents operating in the ecology. Addressing this open question is one of the main objectives of the present study.

**Fig 6:**
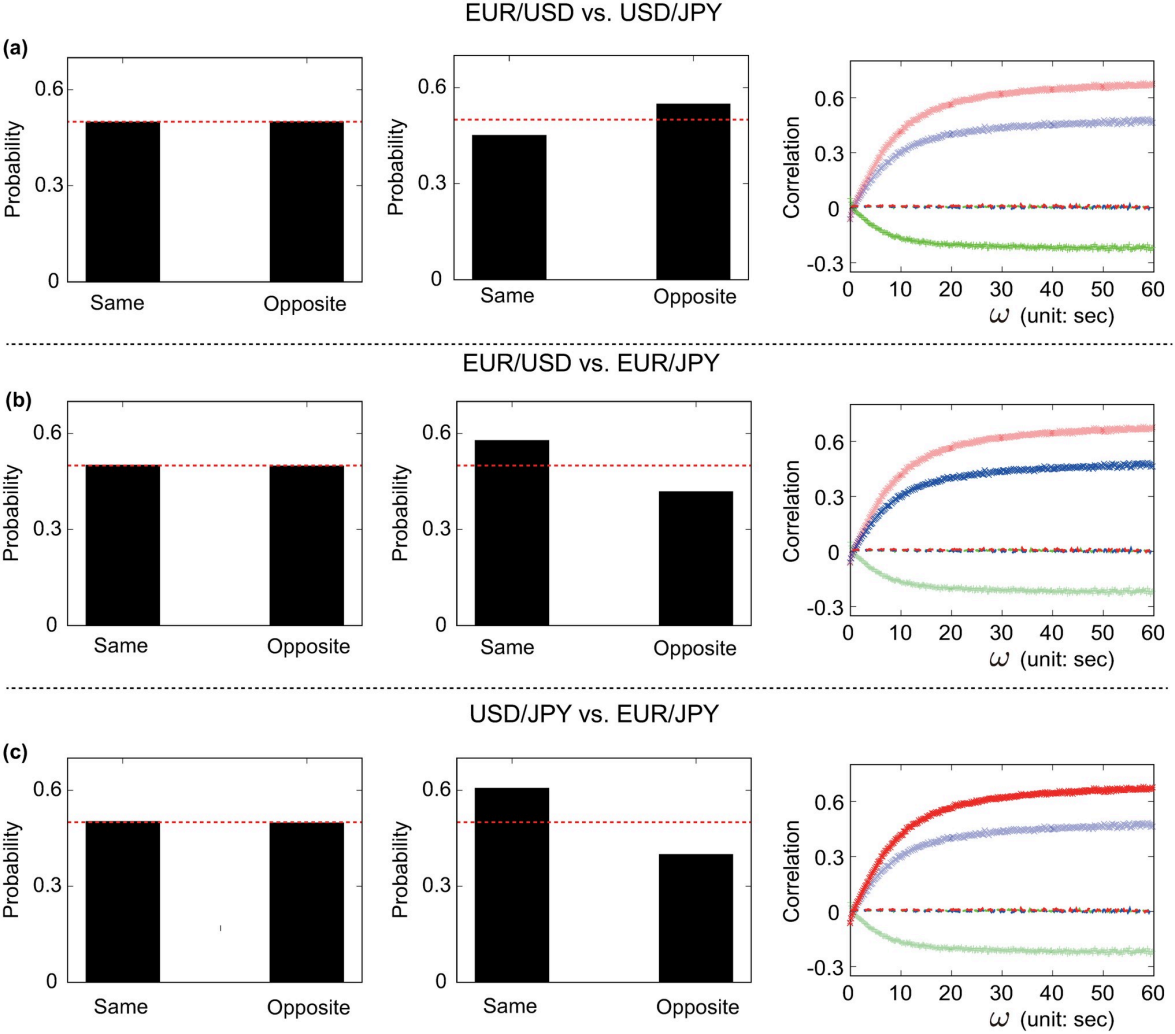
Statistical relationships between different FX markets. Probability of observing two markets in the same or opposite state in the absence of the arbitrager (left column), with the arbitrager (central column) and the associated cross-correlation functions *ρ*_*i*,*j*_(*ω*) (right column) for (a) ΔEUR/USD vs. ΔUSD/JPY, (b) ΔEUR/USD vs. ΔEUR/JPY and (c) ΔUSD/JPY vs. ΔEUR/JPY. The red solid line in the histograms marks the value of 0.5, highlighting the case in which two markets have the same probability of being in the same or opposite state. The lines indicating the value of the cross-correlation function *ρ*_*i*,*j*_(*ω*) are solid (dashed) for experiments including (excluding) the arbitrager. Simulations are performed under the same settings of the experiment presented in Fig 5(b), bottom panel. The inclusion of the arbitrager increases the probability of observing EUR/USD and USD/JPY as well as EUR/USD and EUR/JPY in the same state and USD/JPY and EUR/USD in the opposite state. Furthermore, the active presence of this special agent intertwines the dynamics of different FX rates, creating cross-correlations functions that resemble those emerging in real trading data.

**Fig 7:**
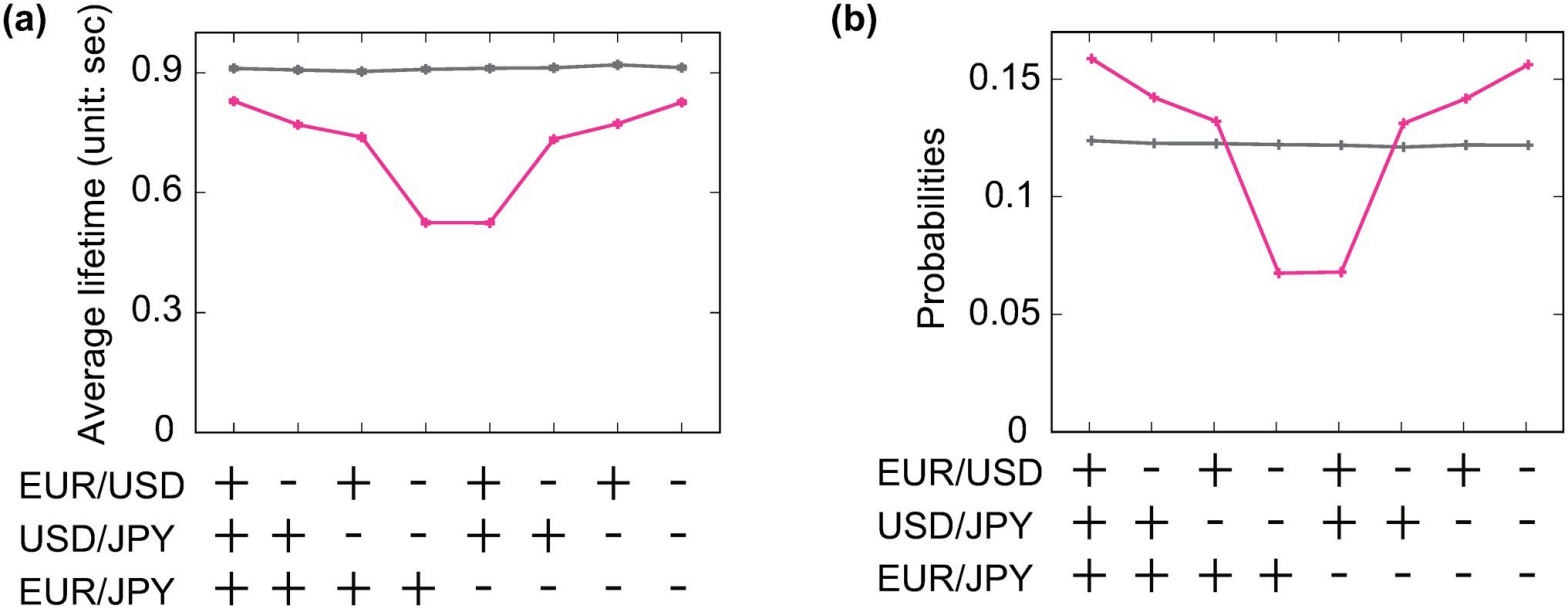
Expected lifetime and appearance probability of the eight ecology configurations. Statistics are collected from simulations of the Arbitrager Model with active (violet) and inactive (grey) arbitrager. Simulations are performed under the same settings of the experiment presented in Fig 5(b), bottom panel. The presence of an active arbitrager increases the average lifetimes (a) and appearance probabilities (b) of certain configurations and reduces the same statistics for others. Statistics in (a) are expressed in real time (i.e., sec.), details on the conversion between simulation time (i.e., time steps) and real time (i.e., sec) are provided in S3.2 Section.

The actual state of the *j*-th market *ν*_*j*_(*t*) is defined as the sign of the current price trend sgn(*ϕ*_*n*,*ℓ*_(*t*)) ∈ {−, +}, see Eq (6). It follows that the current configuration of the ecology *q*(*t*) = {*ν*_1_(*t*), *ν*_2_(*t*), *ν*_3_(*t*)} is the combination of the states of each market. The Arbitrager Model, considering three markets, admits 2^3^ = 8 different ecology configurations. When the arbitrager is not included in the system, two markets have the same probability of being in the same and opposite state, see first column of Fig 6. This occurs because price trends are driven by transactions triggered by endogenous decisions, that is, events occurring in different markets remain completely unrelated. As a consequence, market states flip independently and at the same rate. It follows that the eight possible combinations of market states share the same appearance probabilities 1/2^3^ and expected lifetimes, see Fig 7. In these settings, the dynamics of the mid price of FX rate pairs do not present any significant correlation, see third column of Fig 6.

The inclusion of the arbitrager has a major impact on the overall behavior of the model. Imbalances in the probability of observing two markets in the same or opposite state emerge in each FX rate pair. For instance, the EUR/USD and EUR/JPY markets have the same state in ≈ 57% of the experiment duration, see Fig 6(b). Movements of FX rate pairs become correlated, revealing cross-correlation functions *ρ*_*i*,*j*_(*ω*) whose shapes qualitatively mimic those found in real trading data. The sign and stabilization levels of these functions are consistent with the sign and size of the probabilities imbalances, suggesting that these two results are two faces of the same coin.

The statistical properties of the eight ecology configurations shall be examined in order to understand how the findings presented in Fig 6 unfold. The presence of the arbitrager introduces a degree of heterogeneity in both the expected lifetimes and appearance probabilities of ecology configurations, see Fig 7. This reveals three interesting facts. First, the average lifetime of every ecology configuration is smaller than its counterpart in an arbitrager-free system. To explain this feature, recall that predatory market orders trigger three simultaneous transactions (i.e., one in each market) altering the current price trends *ϕ*_*n*,*ℓ*_(*t*), see Eq (6). When the latest change in transaction price *p*_*ℓ*_(*g*_*t*,*ℓ*_) − *p*_*ℓ*_(*g*_*t*,*ℓ*_ − 1) induced by a predatory market order and *ϕ*_*n*,*ℓ*_(*t* − *dt*) have opposite signs, the actions of the arbitrager weaken (i.e., |*ϕ*_*n*,*ℓ*_(*t*)| < |*ϕ*_*n*,*ℓ*_(*t* − *dt*)|) or even flip the sign (i.e., *ϕ*_*n*,*ℓ*_(*t*)*ϕ*_*n*,*ℓ*_(*t* − *dt*)<0) of the price trend. When this occurs, the arbitrager weakens the trend-following behaviors of market makers in at least one of the three markets, thus increasing the likelihood of a transition to another ecology configuration. As triangular arbitrage opportunities of both types appear, with different incidences, during any ecology configuration, see S15 Fig, the expected lifetimes of these configurations are, to different extents, shorter than in an arbitrager-free system.

Second, certain ecology configurations are expected to last more than others (i.e., single episodes). As reactions to triangular arbitrage opportunities increase the likelihood of flipping a market state, the average lifetime of a given configuration relate to the time required for the first triangular arbitrage opportunity to emerge. For instance, the time between the inception and the first time *μ*^*I*^(*t*) or *μ*^*II*^(*t*) becomes larger than one never exceeds 4 sec for {−, −, +}, which is the configuration with shortest expected lifetime, while it can reach ≈ 6.5 sec for {+, +, +}, which is the configuration with longest expected lifetime, see S14 Fig. This difference can be intuitively explained by looking at the combination of market states. When the ecology configuration is {−, −, +}, EUR/USD and USD/JPY have the opposite state of EUR/JPY. In this scenario, the implied FX cross rate EUR/USD × USD/JPY moves in the opposite direction of the FX rate EUR/JPY, creating the ideal conditions for a rapid emergence of triangular arbitrage opportunities. Conversely, the three markets share the same state when the ecology configuration is {+, +, +}. In this case, both the FX rate and the implied FX cross rate move in the same direction, extending the time required by these prices to create a gap that can be exploited by the arbitrager.

The third and final interesting fact emerged in Fig 7 is that certain configurations are more likely to appear than others. To understand this aspect, consider the significant differences between the probabilities of transitioning from a configuration to another, see S5 Table in S1 File. For instance, assuming that the system is leaving {+, +, +}, the probabilities of transitioning to {−, +, +} and {+, +, −} are 35.8% and 22.7%, respectively. This difference can be explained by the fact that it is much easier to flip the state of EUR/USD and move to {−, +, +} than flipping EUR/JPY and move to {+, +, −}. The value of the price trend *ϕ*_*n*,*ℓ*_(*t*) can be intuitively seen as the *resistance* to state changes of the *ℓ*-th market: the higher its value, the more the transaction price must fluctuate in the opposite direction to flip its sign. For each configuration, the absolute value of this statistics is sampled at the emergence of any triangular arbitrage opportunity. Then, its average is normalized by the initial center of mass *p*_*ℓ*_(*t*_0_), see S3.2 Section, to make it comparable with the same quantity measured in other markets. For {+, +, +}, 〈|*ϕ*_*n*,*ℓ*_(*t*)|〉/*p*_*ℓ*_(*t*_0_) is substantially higher for EUR/JPY than EUR/USD and USD/JPY, see S17 Fig. As a result, predatory market orders are more likely to set the ground for transitions from {+, +, +} to {−, +, +} (35.8%) or {+, −, +} (33.6%). Looking at these transitions on the opposite direction is even more compelling: {+, +, +} is the most likely destination from both {−, +, +} (37.5%) and {+, −, +} (36.4%). This hints at the presence of a loop in which the ecology transits from {+, +, +} to {−, +, +} or {+, −, +} and then moves back. Such dynamics find an explanation in the fact that the market that has recently flipped its state, causing a departure from {+, +, +} towards {−, +, +} or {+, −, +}, can be easily flipped back again before its *resistance* to state changes *ϕ*_*n*,*ℓ*_(*t*) increases in absolute value. This happens when the arbitrager responds to a type 2 triangular arbitrage opportunity (i.e., *μ*^*II*^(*t*)>1) when the ecology configuration is either {−, +, +} or {+, −, +}.

The significant probabilities of returning to {+, +, +} stem from the interplay of two elements. First, triangular arbitrage opportunities are more likely to be of type 2 than type 1 in both {−, +, +} and {+, −, +}, see S15 Fig. Second, the markets with lowest *resistance* to state changes 〈|*ϕ*_*n*,*ℓ*_(*t*)|〉/*p*_*ℓ*_(*t*_0_) are EUR/USD for {−, +, +} and USD/JPY for {+, −, +}, see S17 Fig, which are exactly the states that should be flipped to return to {+, +, +}. The conditional transition probability matrix displayed in S5 Table in S1 File reveals the presence of another configuration triplet (i.e., {−, −, −}, {−, +, −} and {+, −, −}) exhibiting an analogous behavior while {−, −, +} and {+, +, −} are the only two configurations that are not part of any loop. S16 Fig shows this mechanism in action by displaying the sequence of ecology configurations during a segment of the model simulation. It is easy to observe how the system tends to move across configurations belonging to the same looping triplet for long, uninterrupted time windows. Ultimately, this peculiar mechanism increases, to different degrees, the appearance probabilities of configurations involved in these loops at the expenses of {−, −, +} and {+, +, −}.

To sum up, the Arbitrager Model elucidates how the interplay between different trading strategies entangles the dynamics of different FX rates, leading to the characteristic shape of the cross-correlation functions observed in real trading data. The Arbitrager Model restricts its focus to the interactions between two types of strategies, namely triangular arbitrage and trend-following. Despite the simplicity of this framework, the interplay between these two strategies alone satisfactorily reproduces the cross-correlation functions observed in real trading data. In particular, trend-following strategies preserve the current combination of market states for some time while reactions to triangular arbitrage opportunities *influence* the behavior of trend-following market makers by altering the price trend signals used in their dealing strategies. The interactions between these two strategies constantly push the system towards certain configurations and away from others through multiple mechanisms. This can be easily seen in Fig 7 as two distinct statistics, the average expected lifetimes and the appearance probability, put the eight configurations in the same order. For instance {+, +, +} has the longer expected lifetime but also the highest appearance probability. This *force* shapes the features of the statistical relationships between currency pairs. FX rates traded in markets that share the same state in configurations with higher (lower) appearance probabilities and longer (shorter) expected lifetimes are more likely to fluctuate in the same (opposite) direction. For instance, consider USD/JPY and EUR/JPY. These two markets have the same states in the four configurations with higher probabilities (i.e., {+, +, +}, {−, +, +}, {+, −, −} and {−, −, −}) and opposite states in those with lower probabilities (i.e., {+, −, +}, {−, −, +}, {+, +, −} and {−, +, −}). It follows that the probability of observing USD/JPY and EUR/JPY in the same state at a given point in time *t* is ≈ 60%, see Fig 6. In these settings, the mid price dynamics of two FX rates become permanently entangled, leading to the cross-correlation functions displayed in Figs 5(b) and 6.

## 4 Discussion and outlook

The purpose of this study was to obtain further insights into the microscopic origins of the widely documented cross-correlations among currencies. To take up this challenge, a new ABM, the Arbitrager Model, has been proposed as a simple tool to describe the interplay between trend-following and triangular arbitrage strategies across three FX markets. In these settings, the model reproduced the characteristic shape of the cross-correlation function between fluctuations of FX rate pairs under the assumption that triangular arbitrage is the only mechanism through which the different FX rates become synchronized. This suggests that triangular arbitrage plays a primary role in the entanglement of the dynamics of currency pairs in real FX markets. In addition, the model explains how the features of *ρ*_*i*,*j*_(*ω*) emerges from the interplay between triangular arbitrage and trend-following strategies. In particular, triangular arbitrage *influences* the trend-following behaviors of liquidity providers, driving the system towards certain combinations of price trend signs and away from others. This affects the probabilities of observing two FX rates drifting in the same or opposite direction, making one of the two scenarios more likely than the other. Ultimately, this entangles the dynamics of these prices, creating the significant cross-currency correlations that are reproduced in our model and observed in real trading data.

The present study, finding a common ground between previous microscopic ABMs of the FX market and triangular arbitrage [37, 42, 59], sets a new benchmark for further investigations on the relationships between agent interactions and market interdependencies. In particular, it is the first ABM to provide a complete picture on the microscopic origins of cross-currency correlations.

The outcomes of this work open different research paths and raise new challenges that shall be considered in future studies:

- The Arbitrager Model could be further generalized by including a larger number of currencies, allowing traders to monitor different currency triangles. Extending the number of available currencies could reveal new insights into i) statistical regularities related to the triangular arbitrage processes, such the distributions of *μ*^*I*^(*t*) and *μ*^*II*^(*t*), and ii) how the features of the cross-correlation function between two FX rates stem from a much more complex system in which the same FX rate is part of several triangles.
- A potential extension of this model should consider the active presence of special agents operating in FX markets. For instance, simulating public interventions implemented by central banks could be a valuable exercise to understand how the large volumes moved by these entities affect the dynamics of the triangular arbitrage processes *μ*^*I*^(*t*) and *μ*^*II*^(*t*) and the local correlations (i.e., in the intervention time window) between currency pairs.
- Another interesting path leads to market design problems. This study advanced the hypothesis that changes in the stabilization levels of the cross-correlation functions *ρ*_*i*,*j*_(*ω*) might be rooted in the different tick sizes adopted by EBS in the period covered by the employed dataset. Calling for further investigations, an extended version of the present model should examine how different tick sizes affect the correlations between FX rates.
- Future works shall also consider established (e.g., AR family models) and novel [61] tools to exploit further properties of FX rates co-movements. Such investigations might reveal additional statistical relationships whose mechanistic origins can be studied in an augmented version of the Arbitrager Model.

The model introduced in the present study could be subject of meaningful extensions and enhancements aimed to turn this framework into a valuable tool that could be used by exchanges, regulators and market designers. In particular, its simple settings would allow these entities to make predictions on how regulations or design changes could affect the relationships between FX rates and the properties (e.g., frequency, magnitude, duration, etc.) of triangular arbitrage opportunities in a given market. Furthermore, its applicability might attract the attention of other actors operating in the FX market, such as central banks. The ultimate objective of this work and its potential future extensions shall remain the provision of useful means to enhance the understanding of financial market dynamics, assisting the aforementioned entities in conceiving safer and more efficient trading environments.

## Supporting information

S1 Fig (TIF)

S2 Fig (TIF)

S3 Fig (TIF)

S4 Fig (TIF)

S5 Fig (TIF)

S6 Fig (TIF)

S7 Fig (TIF)

S8 Fig (TIF)

S9 Fig (TIF)

S10 Fig (TIF)

S11 Fig (TIF)

S12 Fig (TIF)

S13 Fig (TIF)

S14 Fig (TIF)

S15 Fig (TIF)

S16 Fig (TIF)

S17 Fig (TIF)

S1 File (PDF)

S2 File (TEX)

## References

[1] Buchanan M. It’s a (stylized) fact! Nature Physics. 2011;8(1):3 10.1038/nphys2191

[2] Gould MD, Porter MA, Williams S, McDonald M, Fenn DJ, Howison SD. Limit order books. Quantitative Finance. 2013;13(11):1709–1742. 10.1080/14697688.2013.803148

[3] Gopikrishnan P, Meyer M, Amaral LN, Stanley HE. Inverse cubic law for the distribution of stock price variations. The European Physical Journal B-Condensed Matter and Complex Systems. 1998;3(2):139–140. 10.1007/s100510050292

[4] Cont R. Empirical properties of asset returns: stylized facts and statistical issues. Quantitative Finance. 2001;1(2). 10.1080/713665670

[5] Plerou V, Stanley HE. Stock return distributions: tests of scaling and universality from three distinct stock markets. Physical Review E. 2008;77(3):037101 10.1103/PhysRevE.77.037101 18517560

[6] Chakraborti A, Toke IM, Patriarca M, Abergel F. Econophysics review: I. Empirical facts. Quantitative Finance. 2011;11(7):991–1012. 10.1080/14697688.2010.539248

[7] Liu Y, Cizeau P, Meyer M, Peng CK, Stanley HE. Correlations in economic time series. Physica A: Statistical Mechanics and its Applications. 1997;245(3-4):437–440. 10.1016/S0378-4371(97)00368-3

[8] Cont R. Volatility clustering in financial markets: empirical facts and agent-based models In: Long memory in economics. Springer; 2007 p. 289–309.

[9] Stanley HE, Plerou V, Gabaix X. A statistical physics view of financial fluctuations: Evidence for scaling and universality. Physica A: Statistical Mechanics and its Applications. 2008;387(15):3967–3981. 10.1016/j.physa.2008.01.093

[10] Gu GF, Zhou WX. Emergence of long memory in stock volatility from a modified Mike-Farmer model. EPL (Europhysics Letters). 2009;86(4):48002 10.1209/0295-5075/86/48002

[11] Zovko I, Farmer JD, et al The power of patience: a behavioural regularity in limit-order placement. Quantitative finance. 2002;2(5):387–392. 10.1088/1469-7688/2/5/308

[12] Lillo F, Farmer JD. The long memory of the efficient market. Studies in nonlinear dynamics & econometrics. 2004;8(3).

[13] Bouchaud JP, Gefen Y, Potters M, Wyart M. Fluctuations and response in financial markets: the subtle nature of ‘random’price changes. Quantitative Finance. 2004;4(2):176–190. 10.1080/14697680400000022

[14] Cont R. Long range dependence in financial markets In: Fractals in engineering. Springer; 2005 p. 159–179.

[15] Zhao L. A model of limit-order book dynamics and a consistent estimation procedure. Citeseer; 2010.

[16] Aït-Sahalia Y, Mykland PA, Zhang L. Ultra high frequency volatility estimation with dependent microstructure noise. Journal of Econometrics. 2011;160(1):160–175. 10.1016/j.jeconom.2010.03.028

[17] Farmer JD, Shubik M, Smith E. Is economics the next physical science? Physics today. 2005;58(9):37–42. 10.1063/1.2117821

[18] Parlour CA, Seppi DJ. Limit order markets: A survey. Handbook of financial intermediation and banking. 2008;5:63–95. 10.1016/B978-044451558-2.50007-6

[19] Chakravarty S, Holden CW. An integrated model of market and limit orders. Journal of Financial Intermediation. 1995;4(3):213–241. 10.1006/jfin.1995.1010

[20] Parlour CA. Price dynamics in limit order markets. The Review of Financial Studies. 1998;11(4):789–816. 10.1093/rfs/11.4.789

[21] Foucault T. Order flow composition and trading costs in a dynamic limit order market1. Journal of Financial Markets. 1999;2(2):99–134. 10.1016/S1386-4181(98)00012-3

[22] Glosten LR, Milgrom PR. Bid, ask and transaction prices in a specialist market with heterogeneously informed traders. Journal of Financial Economics. 1985;14(1):71–100. 10.1016/0304-405X(85)90044-3

[23] Kyle AS. Continuous auctions and insider trading. Econometrica: Journal of the Econometric Society. 1985; p. 1315–1335. 10.2307/1913210

[24] Goettler R, Parlour C, Rajan U, et al. Microstructure effects and asset pricing. Preprint, available at http://en.scientificcommons.org/33345856. 2006;.

[25] Roşu I. A dynamic model of the limit order book. The Review of Financial Studies. 2009;22(11):4601–4641. 10.1093/rfs/hhp011

[26] Rosu I, et al Liquidity and information in order driven markets. Chicago Booth School of Business 2010;.

[27] Bertsimas D, Lo AW. Optimal control of execution costs. Journal of Financial Markets. 1998;1(1):1–50. 10.1016/S1386-4181(97)00012-8

[28] Almgren R, Chriss N. Optimal execution of portfolio transactions. Journal of Risk. 2001;3:5–40. 10.21314/JOR.2001.041

[29] Alfonsi A, Fruth A, Schied A. Optimal execution strategies in limit order books with general shape functions. Quantitative Finance. 2010;10(2):143–157. 10.1080/14697680802595700

[30] Obizhaeva AA, Wang J. Optimal trading strategy and supply/demand dynamics. Journal of Financial Markets. 2013;16(1):1–32. 10.1016/j.finmar.2012.09.001

[31] Anderson P, Arrow K, Pines D. The economy as an evolving complex system Addison-Wesley Redwood City 1988;.

[32] Takayasu H, Miura H, Hirabayashi T, Hamada K. Statistical properties of deterministic threshold elements—the case of market price. Physica A: Statistical Mechanics and its Applications. 1992;184(1-2):127–134. 10.1016/0378-4371(92)90161-I

[33] Sato AH, Takayasu H. Dynamic numerical models of stock market price: from microscopic determinism to macroscopic randomness. Physica A: Statistical Mechanics and its Applications. 1998;250(1-4):231–252. 10.1016/S0378-4371(97)00569-4

[34] Cont R, Bouchaud JP. Herd behavior and aggregate fluctuations in financial markets. Macroeconomic Dynamics. 2000;4(2):170–196. 10.1017/S1365100500015029

[35] Chiarella C, Iori G, et al A simulation analysis of the microstructure of double auction markets*. Quantitative Finance. 2002;2(5):346–353. 10.1088/1469-7688/2/5/303

[36] Challet D, Stinchcombe R, et al Non-constant rates and over-diffusive prices in a simple model of limit order markets. Quantitative Finance. 2003;3(3):155–162. 10.1088/1469-7688/3/3/301

[37] Aiba Y, Hatano N. A microscopic model of triangular arbitrage. Physica A: Statistical Mechanics and its Applications. 2006;371(2):572–584. 10.1016/j.physa.2006.05.046

[38] Preis T, Golke S, Paul W, Schneider JJ. Multi-agent-based order book model of financial markets. EPL (Europhysics Letters). 2006;75(3):510 10.1209/epl/i2006-10139-0

[39] Preis T, Golke S, Paul W, Schneider JJ. Statistical analysis of financial returns for a multiagent order book model of asset trading. Physical Review E. 2007;76(1):016108 10.1103/PhysRevE.76.016108 17677534

[40] Lillo F. Limit order placement as an utility maximization problem and the origin of power law distribution of limit order prices. The European Physical Journal B. 2007;55(4):453–459. 10.1140/epjb/e2007-00067-9

[41] Yamada K, Takayasu H, Takayasu M. Characterization of foreign exchange market using the threshold-dealer-model. Physica A: Statistical Mechanics and its Applications. 2007;382(1):340–346. 10.1016/j.physa.2007.02.027

[42] Yamada K, Takayasu H, Ito T, Takayasu M. Solvable stochastic dealer models for financial markets. Physical Review E. 2009;79(5):051120 10.1103/PhysRevE.79.051120 19518429

[43] Lee S, Lee K. Heterogeneous expectations leading to bubbles and crashes in asset markets: Tipping point, herding behavior and group effect in an agent-based model. Journal of Open Innovation: Technology, Market, and Complexity. 2015;1(1):12 10.1186/s40852-015-0013-9

[44] Cocco L, Concas G, Marchesi M. Using an artificial financial market for studying a cryptocurrency market. Journal of Economic Interaction and Coordination. 2017;12(2):345–365. 10.1007/s11403-015-0168-2

[45] Rime D, Schrimpf A. The anatomy of the global FX market through the lens of the 2013 Triennial Survey. BIS Quarterly Review, December. 2013;.

[46] Bank for International Settlements (BIS). Monitoring of fast-paced electronic markets, Report submitted by a Study Group established by the Market Committee; 2018. Available from: https://www.bis.org/publ/mktc10.pdf.

[47] Mizuno T, Kurihara S, Takayasu M, Takayasu H. Time-scale dependence of correlations among foreign currencies In: The Application of Econophysics. Springer; 2004 p. 24–29. 10.1007/978-4-431-53947-6_3

[48] Wang GJ, Xie C, Chen YJ, Chen S. Statistical properties of the foreign exchange network at different time scales: evidence from detrended cross-correlation coefficient and minimum spanning tree. Entropy. 2013;15(5):1643–1662. 10.3390/e15051643

[49] Han MF, Westelius MNJ. Anatomy of Sudden Yen Appreciations. International Monetary Fund; 2019.

[50] Aiba Y, Hatano N, Takayasu H, Marumo K, Shimizu T. Triangular arbitrage as an interaction among foreign exchange rates. Physica A: Statistical Mechanics and its Applications. 2002;310(3-4):467–479. 10.1016/S0378-4371(02)00799-9

[51] Fenn DJ, Howison SD, McDonald M, Williams S, Johnson NF. The mirage of triangular arbitrage in the spot foreign exchange market. International Journal of Theoretical and Applied Finance. 2009;12(08):1105–1123. 10.1142/S0219024909005609

[52] Kozhan R, Tham WW. Execution risk in high-frequency arbitrage. Management Science. 2012;58(11):2131–2149. 10.1287/mnsc.1120.1541

[53] Foucault T, Kozhan R, Tham WW. Toxic arbitrage. The Review of Financial Studies. 2016;30(4):1053–1094. 10.1093/rfs/hhw103

[54] Bonart J, Lillo F. A continuous and efficient fundamental price on the discrete order book grid. Physica A: Statistical Mechanics and its Applications. 2018;503:698–713. 10.1016/j.physa.2018.03.002

[55] Marshall B, Treepongkaruna S, Young M. Exploitable arbitrage opportunities exist in the foreign exchange market. American Finance Association Annual Meeting, New Orleans. 2008;.

[56] Mahmoodzadeh S, Gençay R. Tick size change in the wholesale foreign exchange market; 2014.

[57] CME Group. EBS; 2019. Available from: https://www.cmegroup.com/trading/market-tech-and-data-services/ebs.html.

[58] CME Group. EBS Platforms; 2019. Available from: https://www.cmegroup.com/trading/market-tech-and-data-services/ebs/platforms.html.

[59] Kanazawa K, Sueshige T, Takayasu H, Takayasu M. Derivation of the Boltzmann equation for financial Brownian motion: Direct observation of the collective motion of high-frequency traders. Physical Review Letters. 2018;120(13):138301 10.1103/PhysRevLett.120.138301 29694225

[60] Bonabeau E. Agent-based modeling: Methods and techniques for simulating human systems. Proceedings of the National Academy of Sciences. 2002;99(suppl 3):7280–7287. 10.1073/pnas.082080899 PMC128598 12011407

[61] Ramos-Requena JP, Trinidad-Segovia JE, Sánchez-Granero MÁ. An Alternative Approach to Measure Co-Movement between Two Time Series. Mathematics. 2020;8(2):261.

