# Supplementary figures and images for: The microscopic relationships between triangular arbitrage and cross-currency correlations in a simple agent based model of foreign exchange markets
Source: PLoS One. 2020 Jun 24;15(6):e0234709. doi: 10.1371/journal.pone.0234709 (PMC7313750; doi:10.1371/journal.pone.0234709)

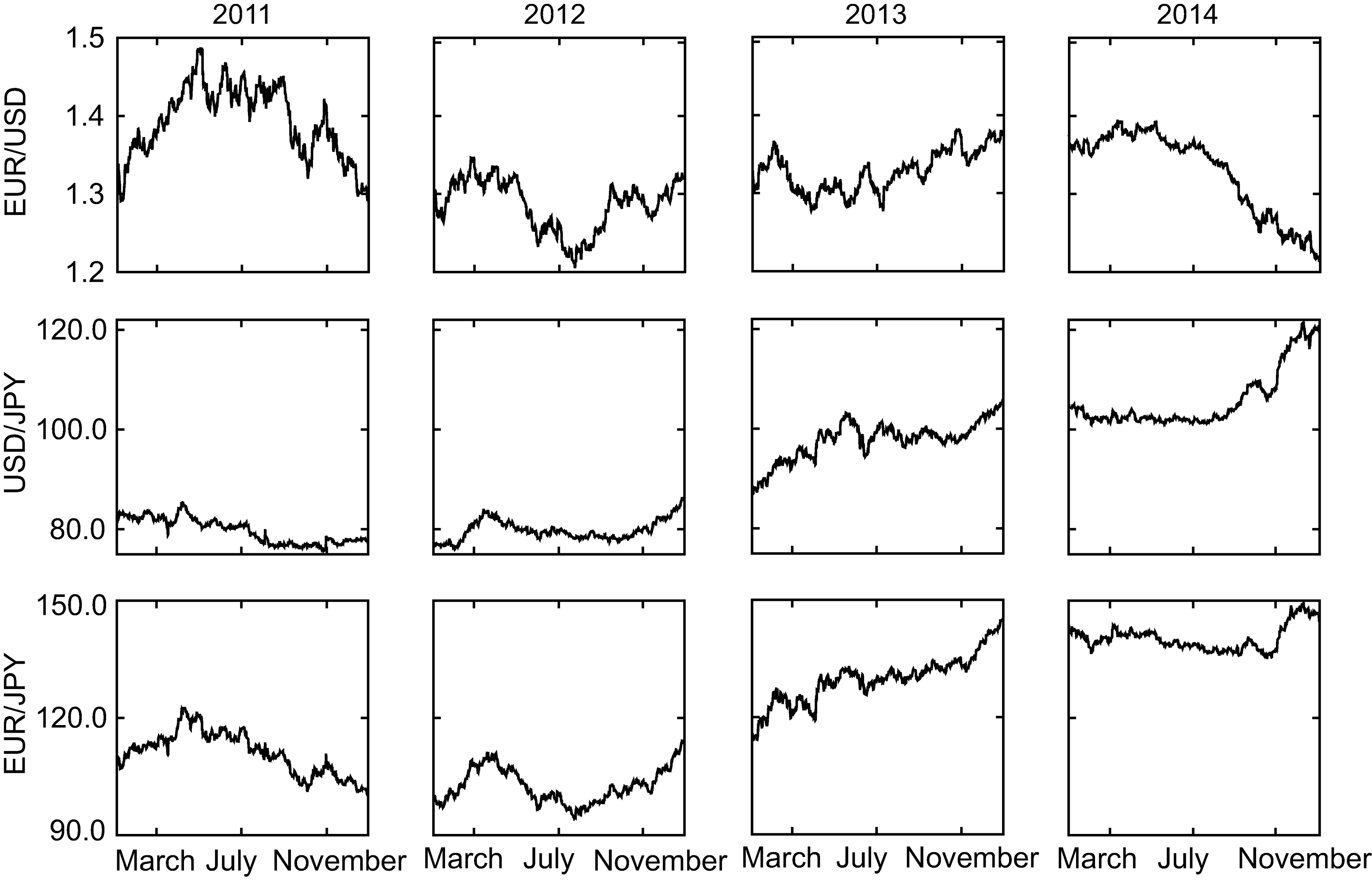

Supplement: S1 Fig — (TIF) [file pone.0234709.s001.tif]

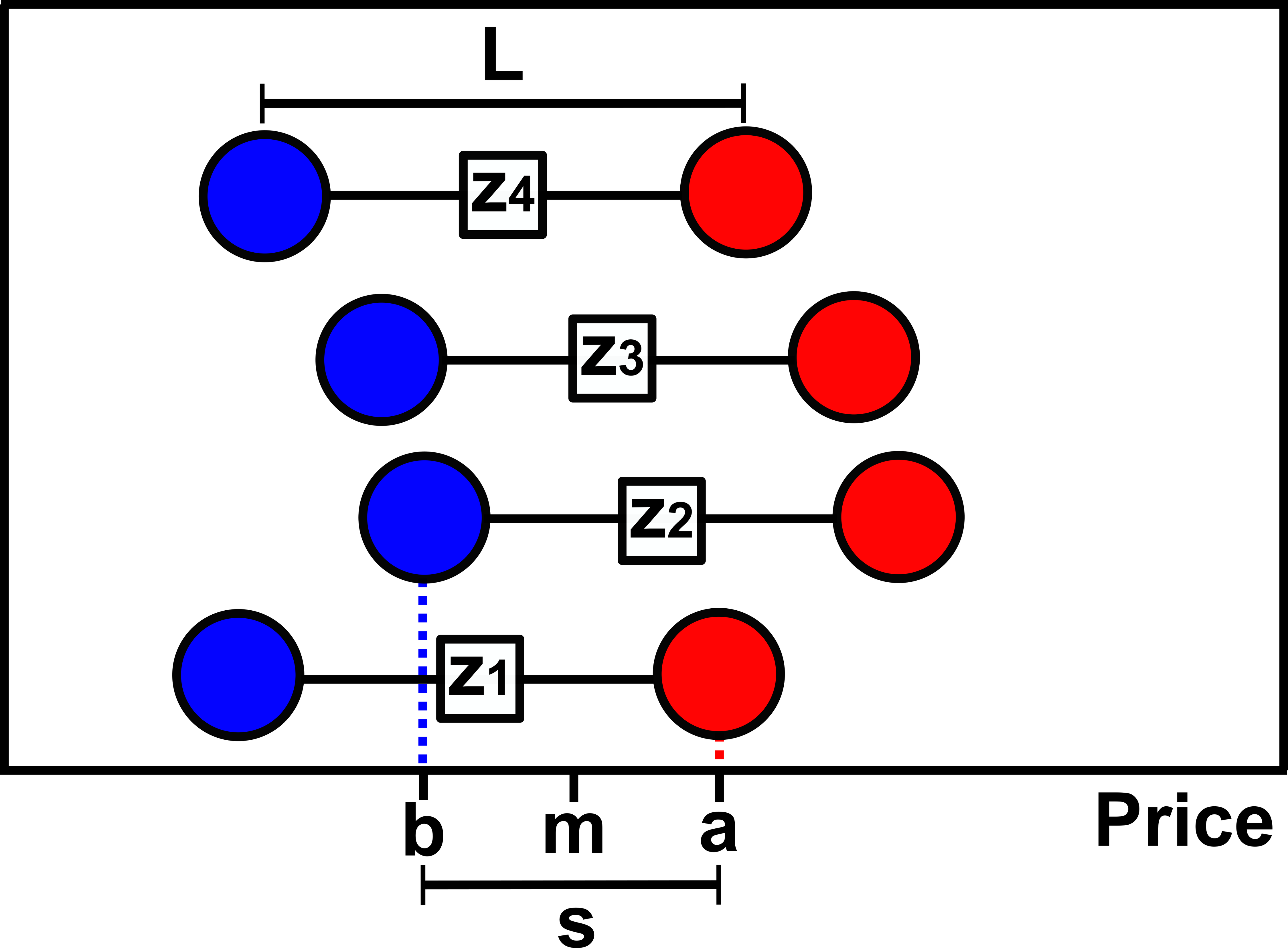

Supplement: S2 Fig — (TIF) [file pone.0234709.s002.tif]

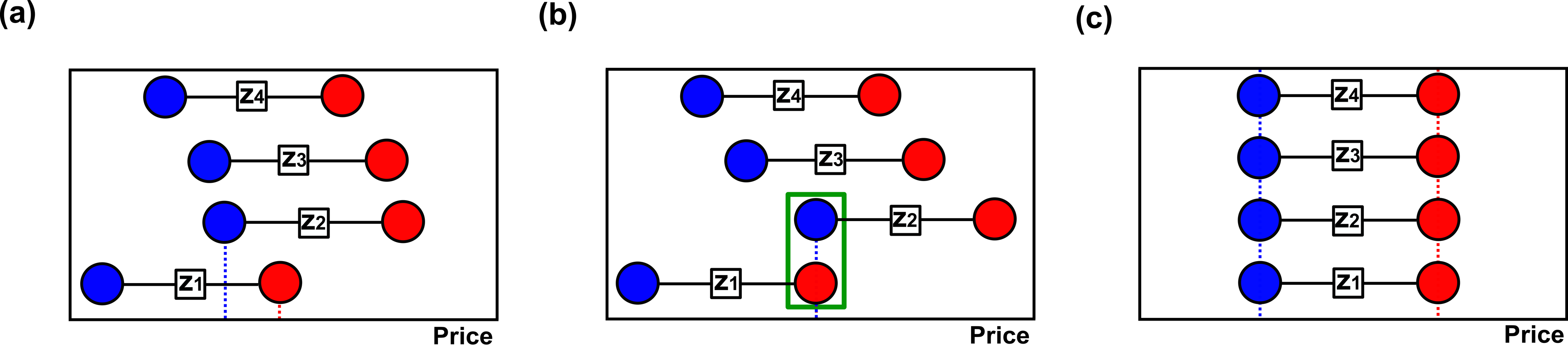

Supplement: S3 Fig — (TIF) [file pone.0234709.s003.tif]

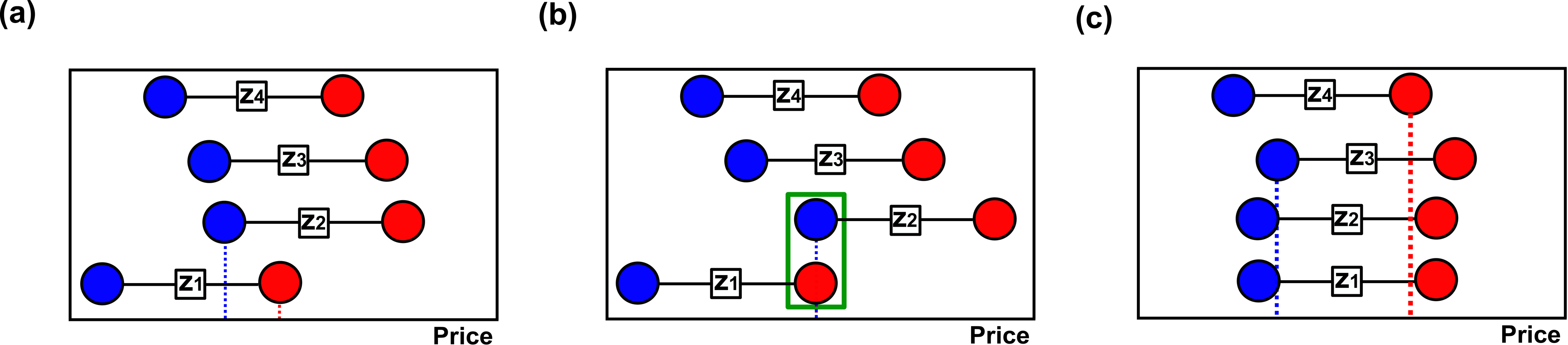

Supplement: S4 Fig — (TIF) [file pone.0234709.s004.tif]

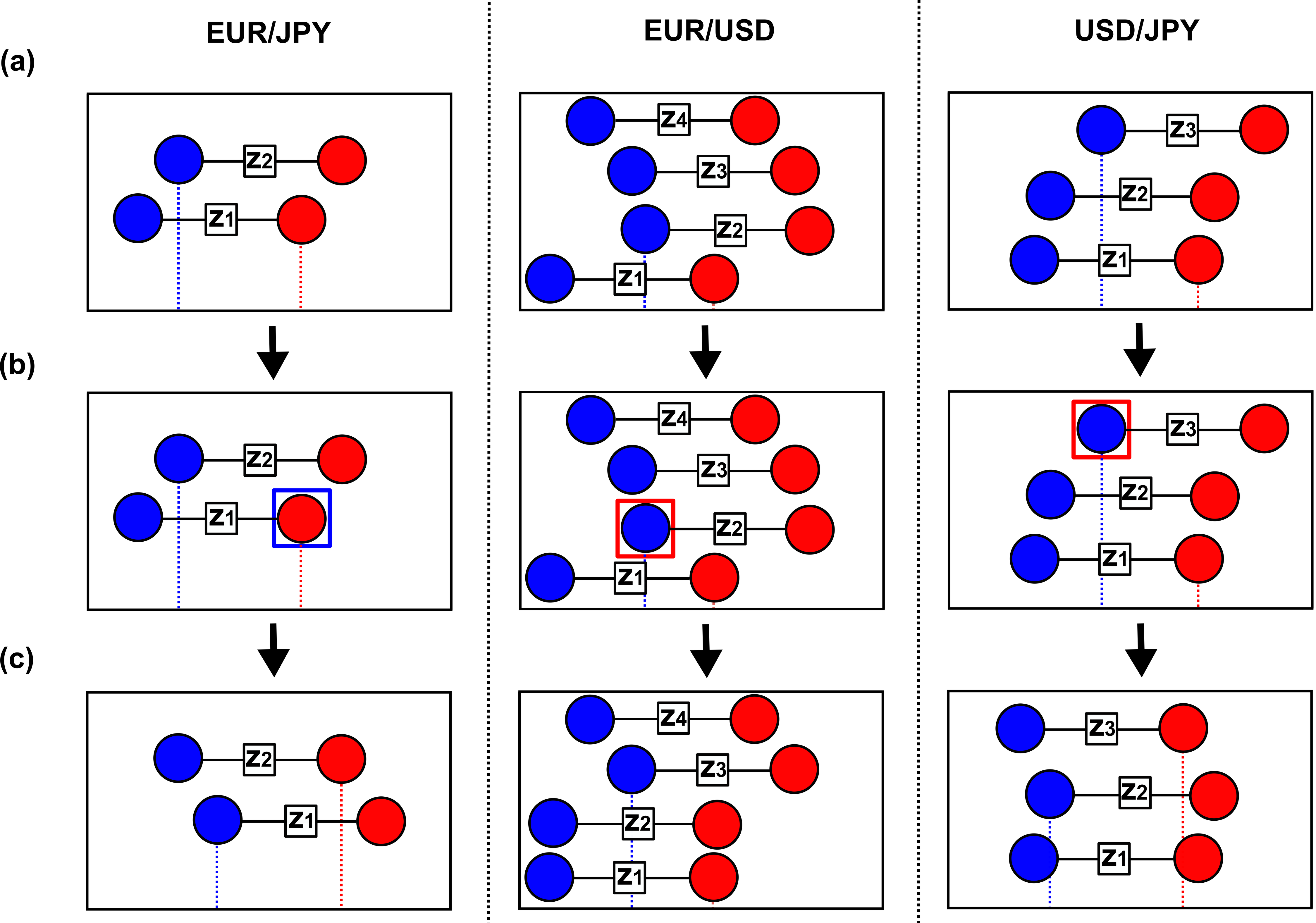

Supplement: S5 Fig — (TIF) [file pone.0234709.s005.tif]

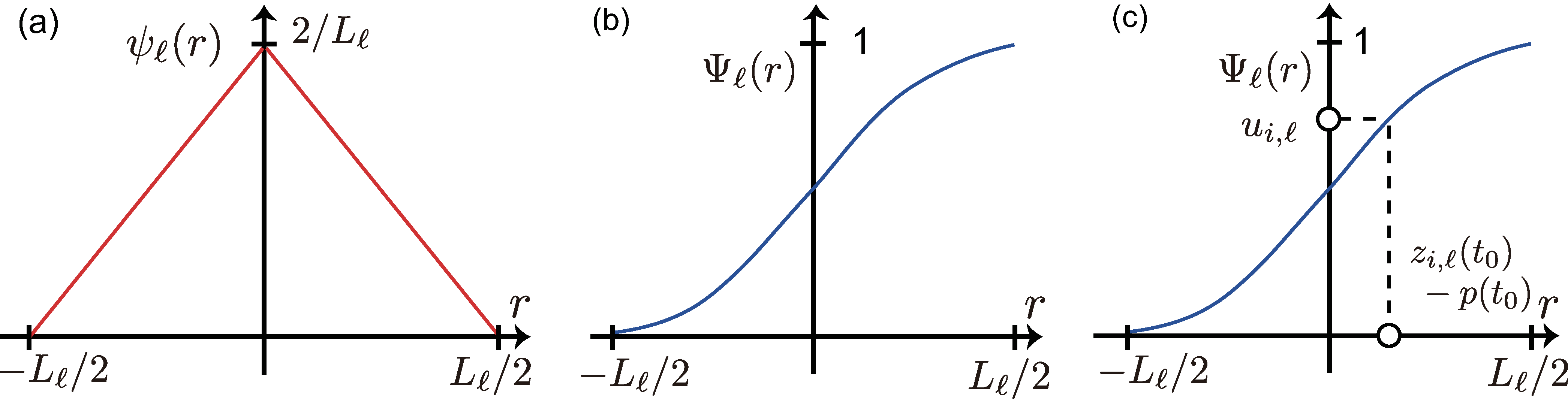

Supplement: S6 Fig — (TIF) [file pone.0234709.s006.tif]

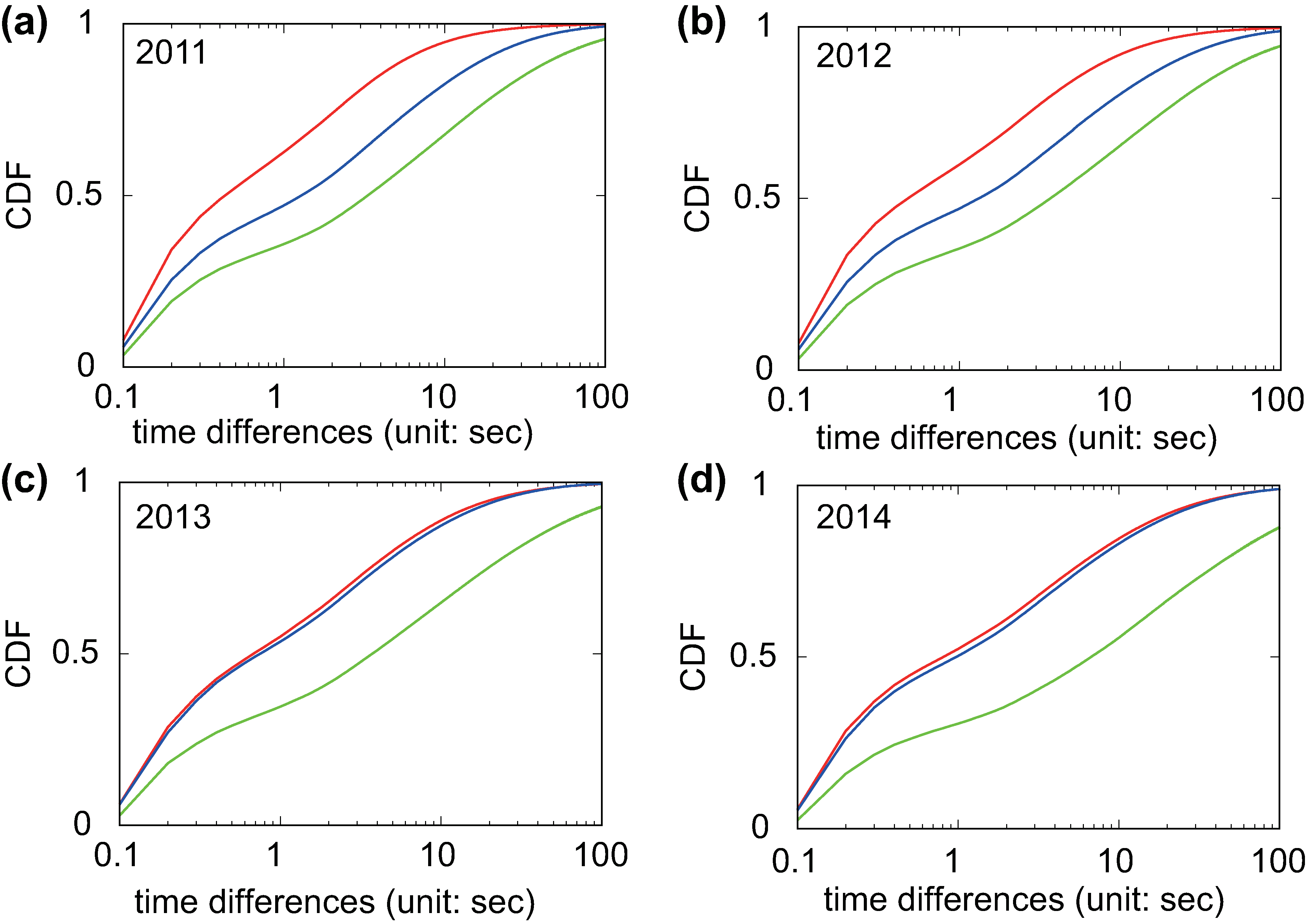

Supplement: S7 Fig — (TIF) [file pone.0234709.s007.tif]

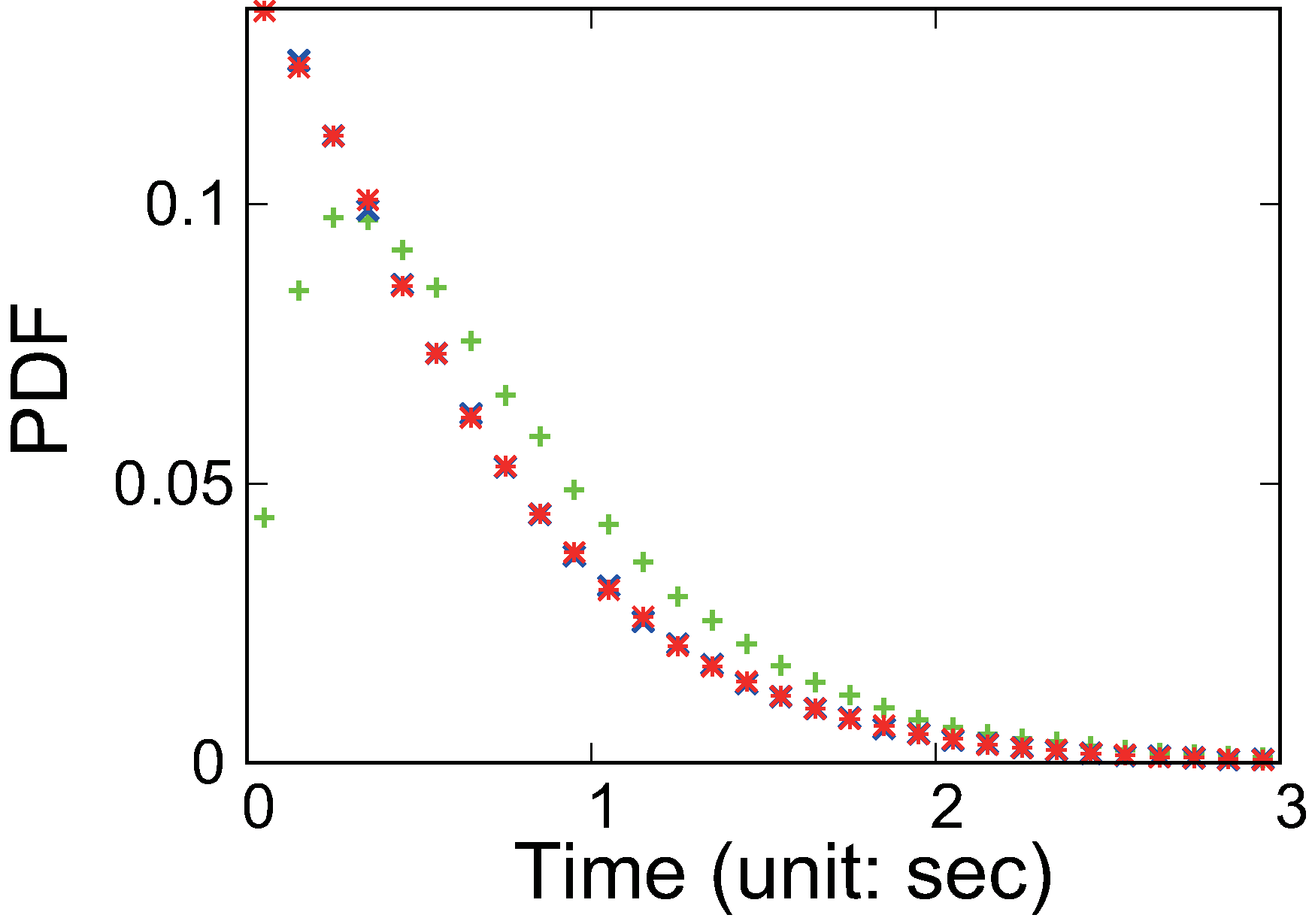

Supplement: S8 Fig — (TIF) [file pone.0234709.s008.tif]

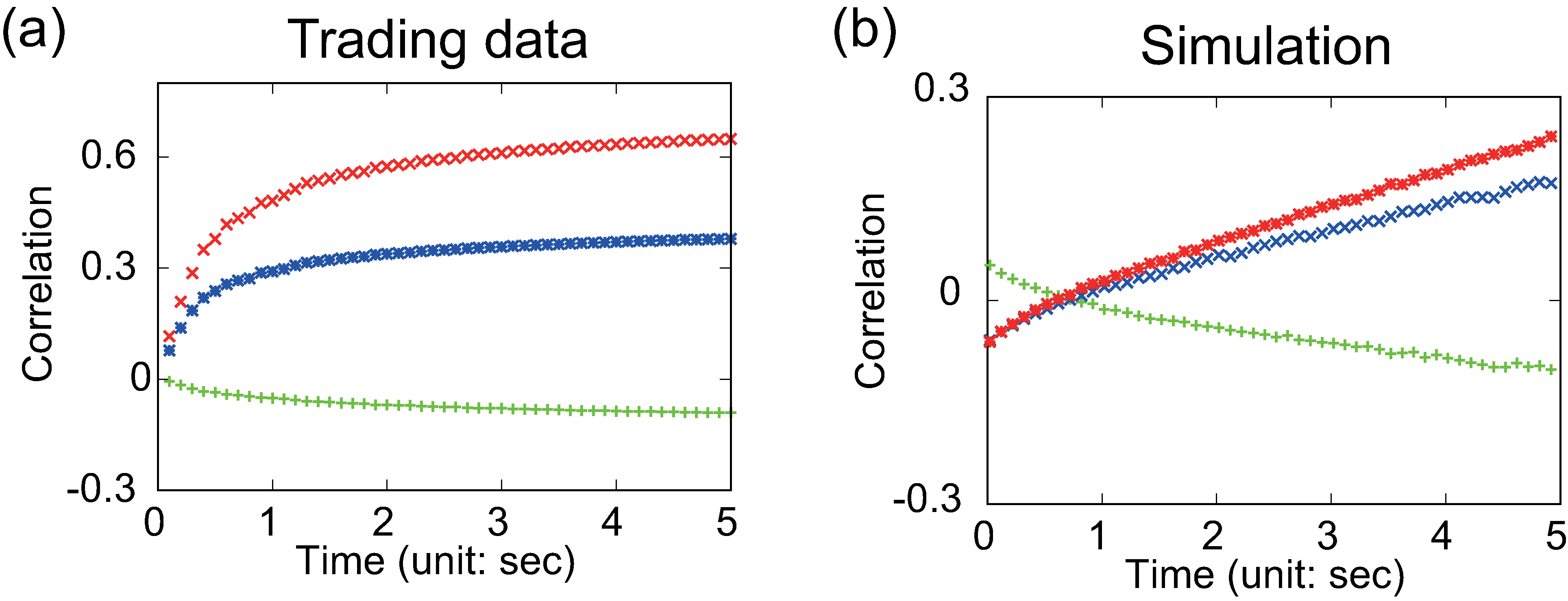

Supplement: S9 Fig — (TIF) [file pone.0234709.s009.tif]

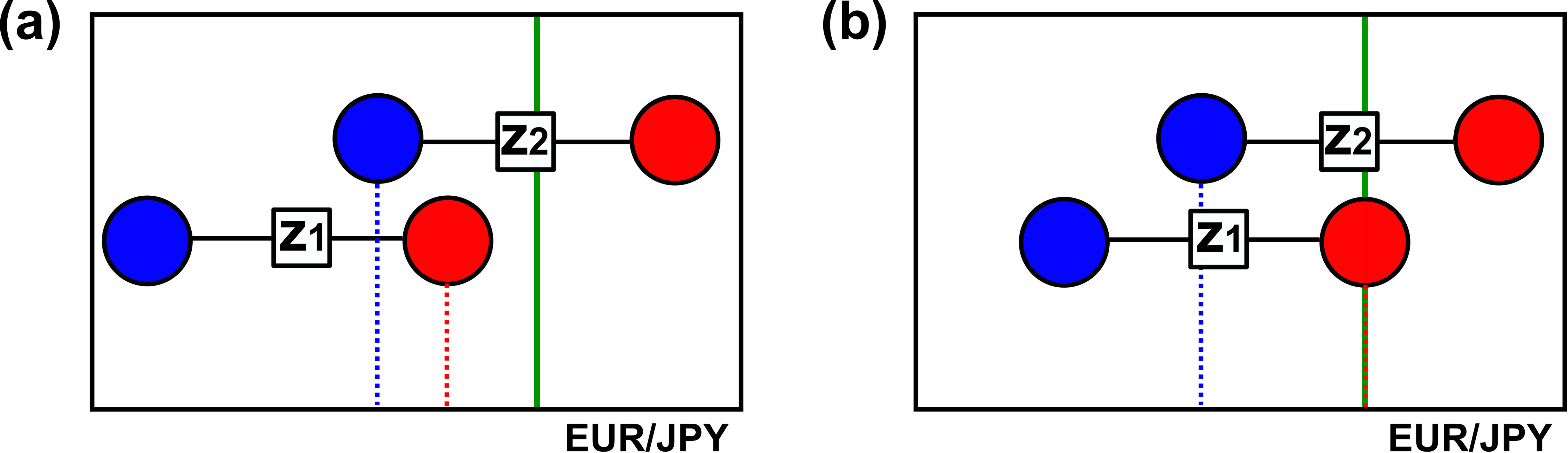

Supplement: S10 Fig — (TIF) [file pone.0234709.s010.tif]

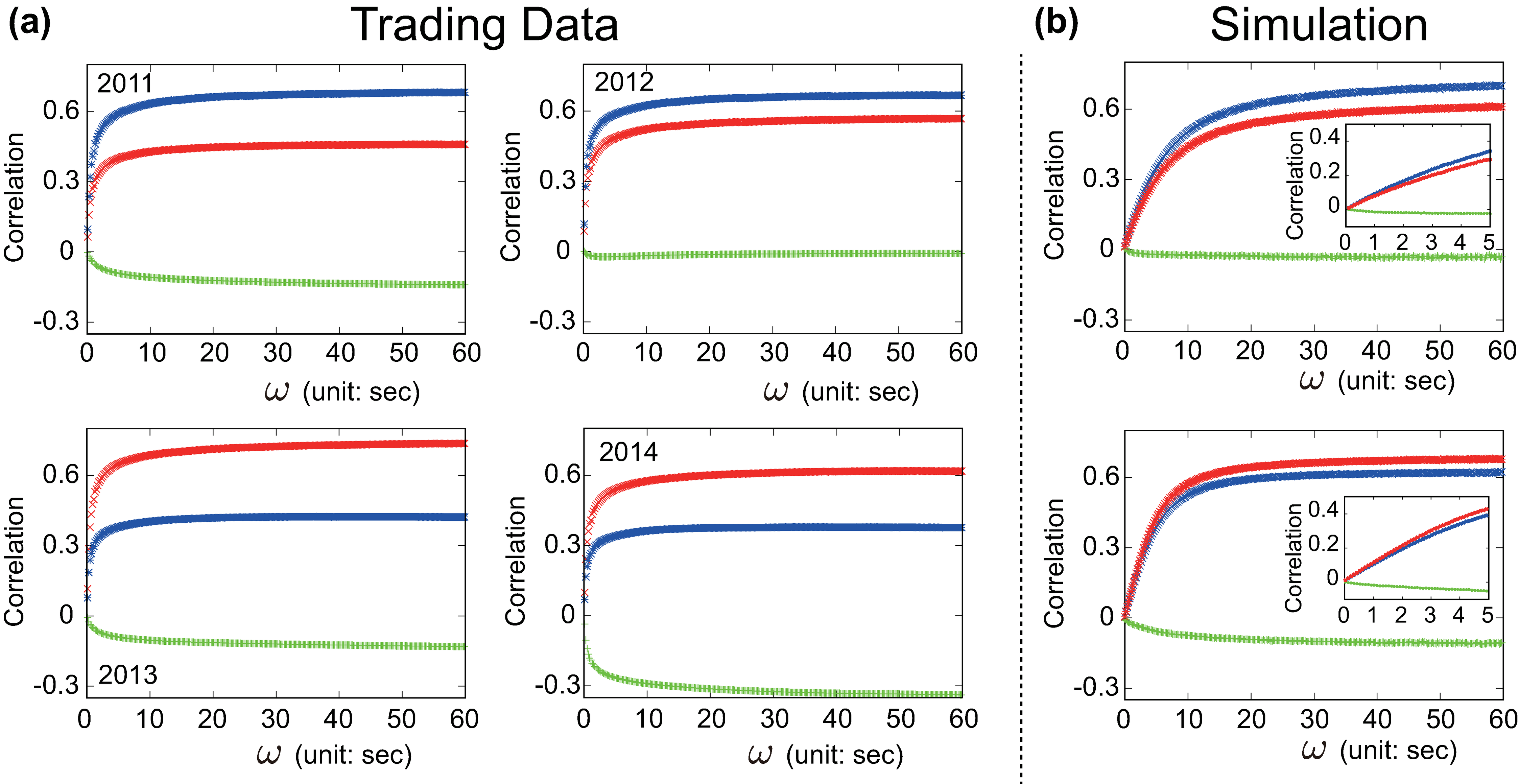

Supplement: S11 Fig — (TIF) [file pone.0234709.s011.tif]

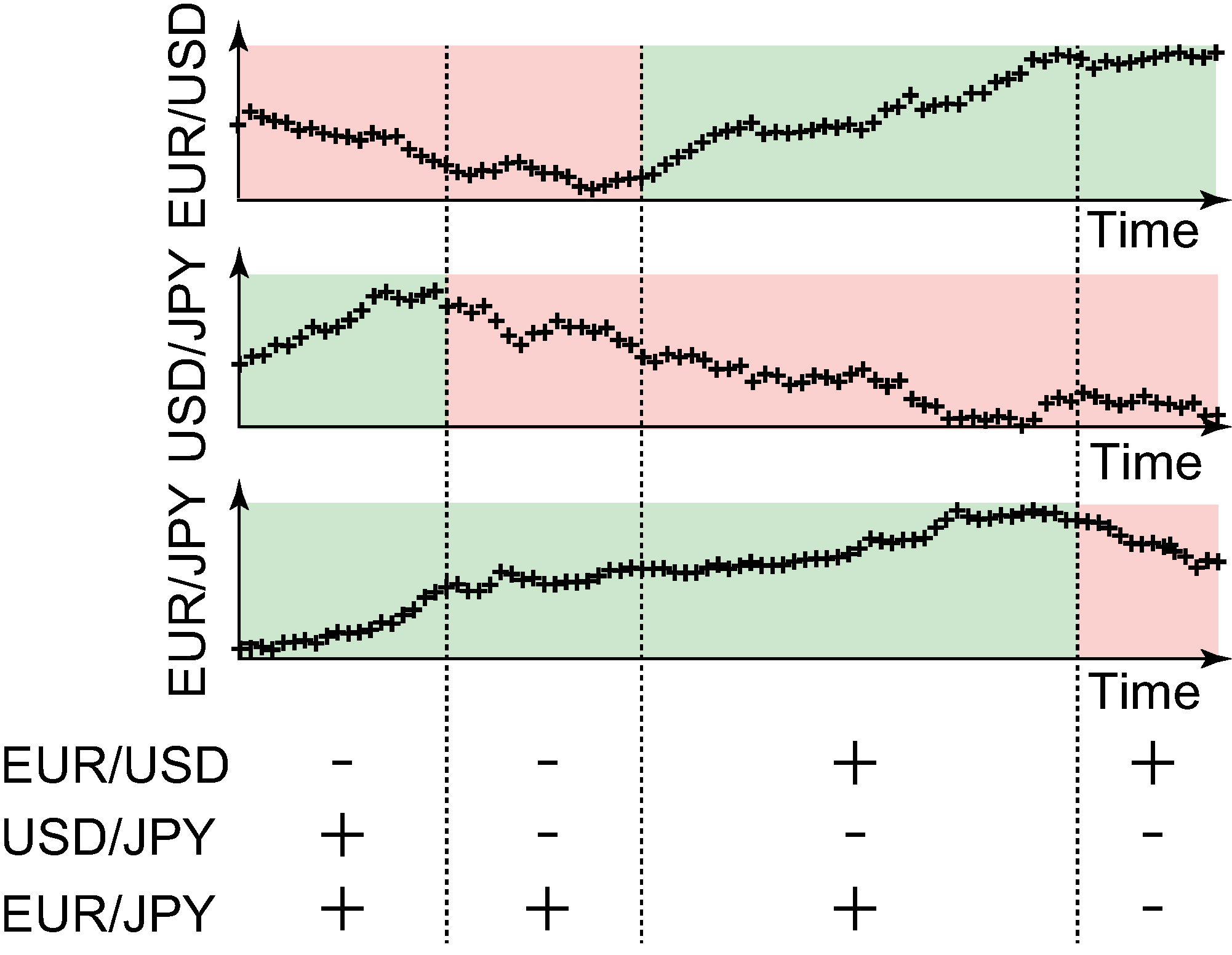

Supplement: S12 Fig — (TIF) [file pone.0234709.s012.tif]

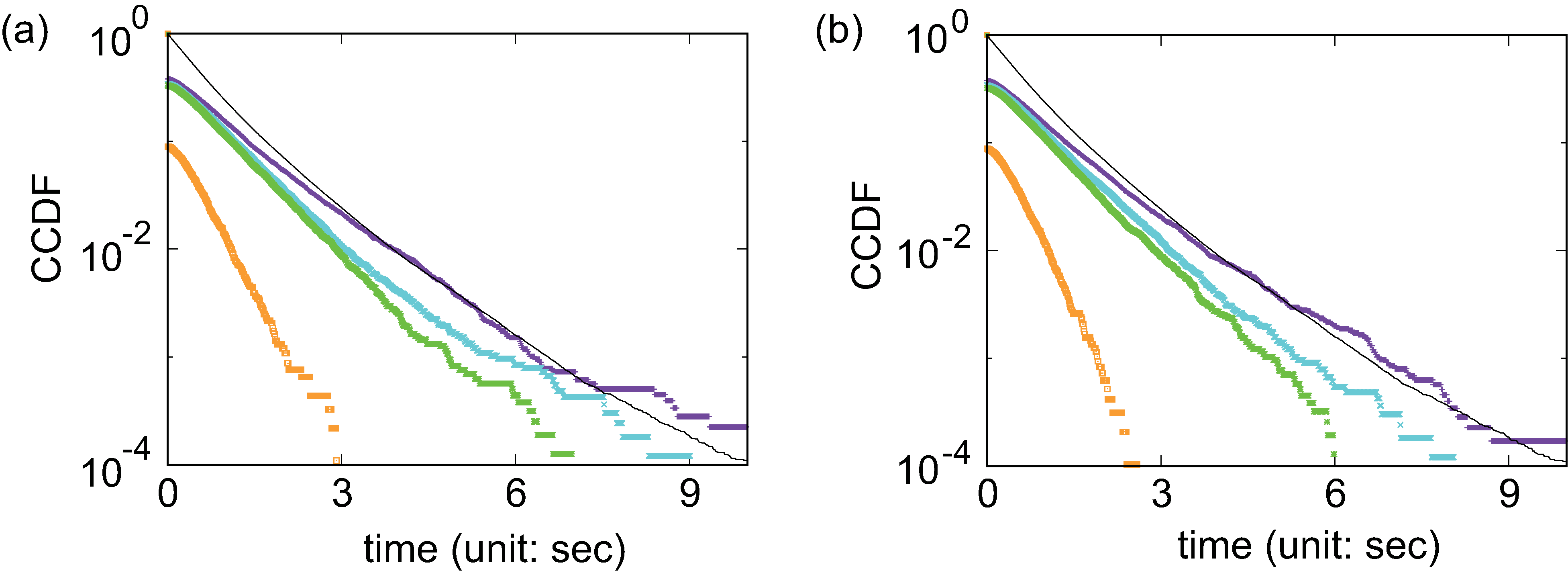

Supplement: S13 Fig — (TIF) [file pone.0234709.s013.tif]

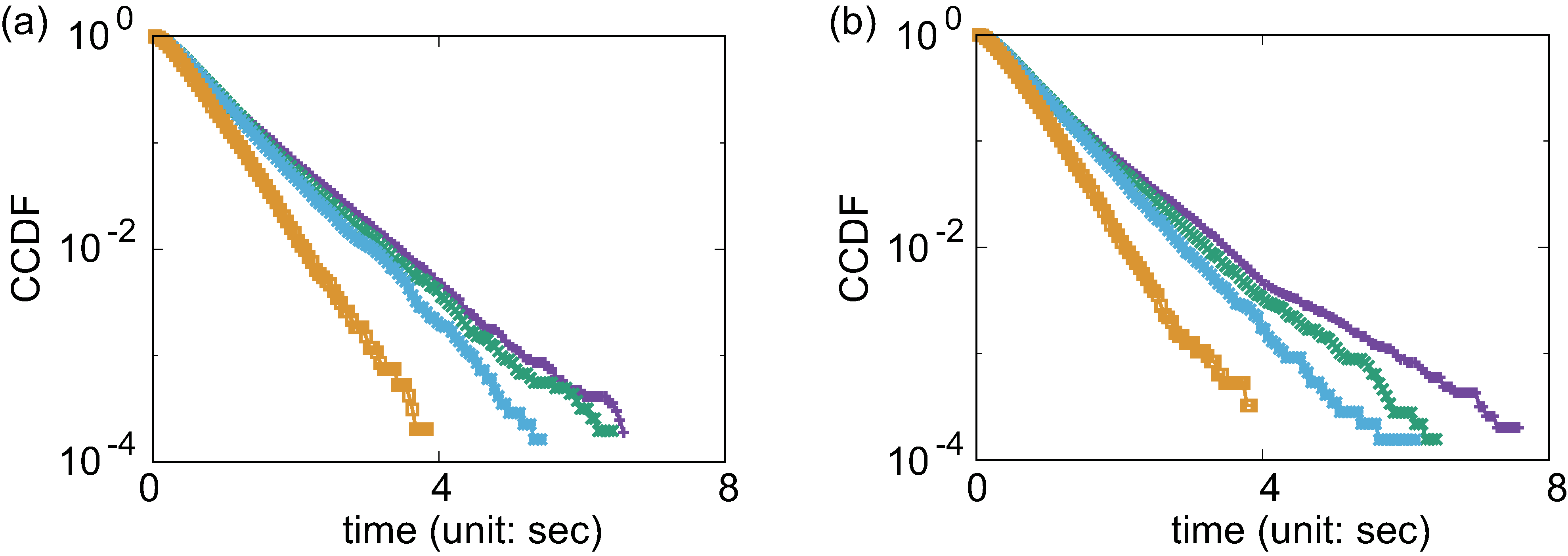

Supplement: S14 Fig — (TIF) [file pone.0234709.s014.tif]

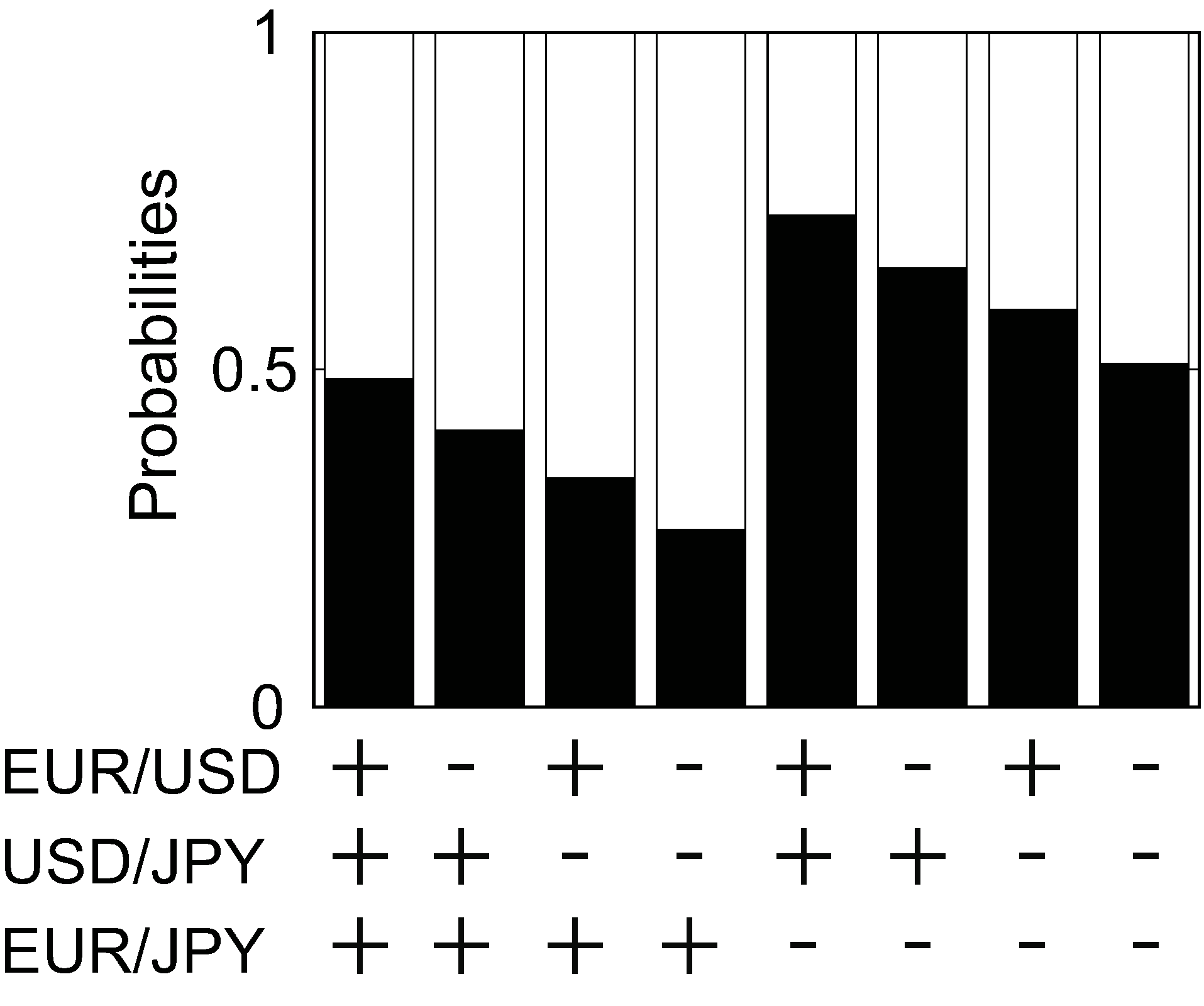

Supplement: S15 Fig — (TIF) [file pone.0234709.s015.tif]

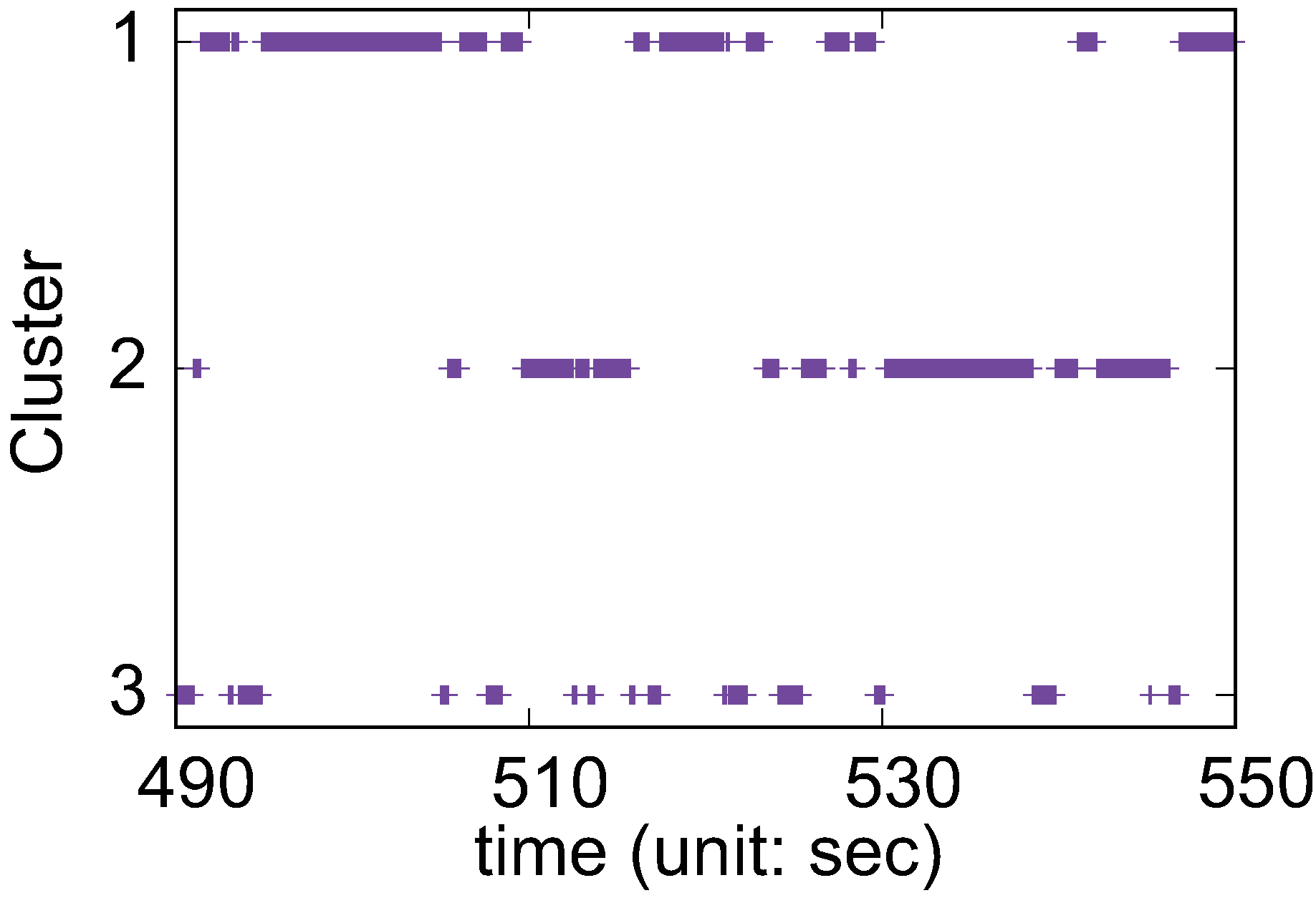

Supplement: S16 Fig — (TIF) [file pone.0234709.s016.tif]

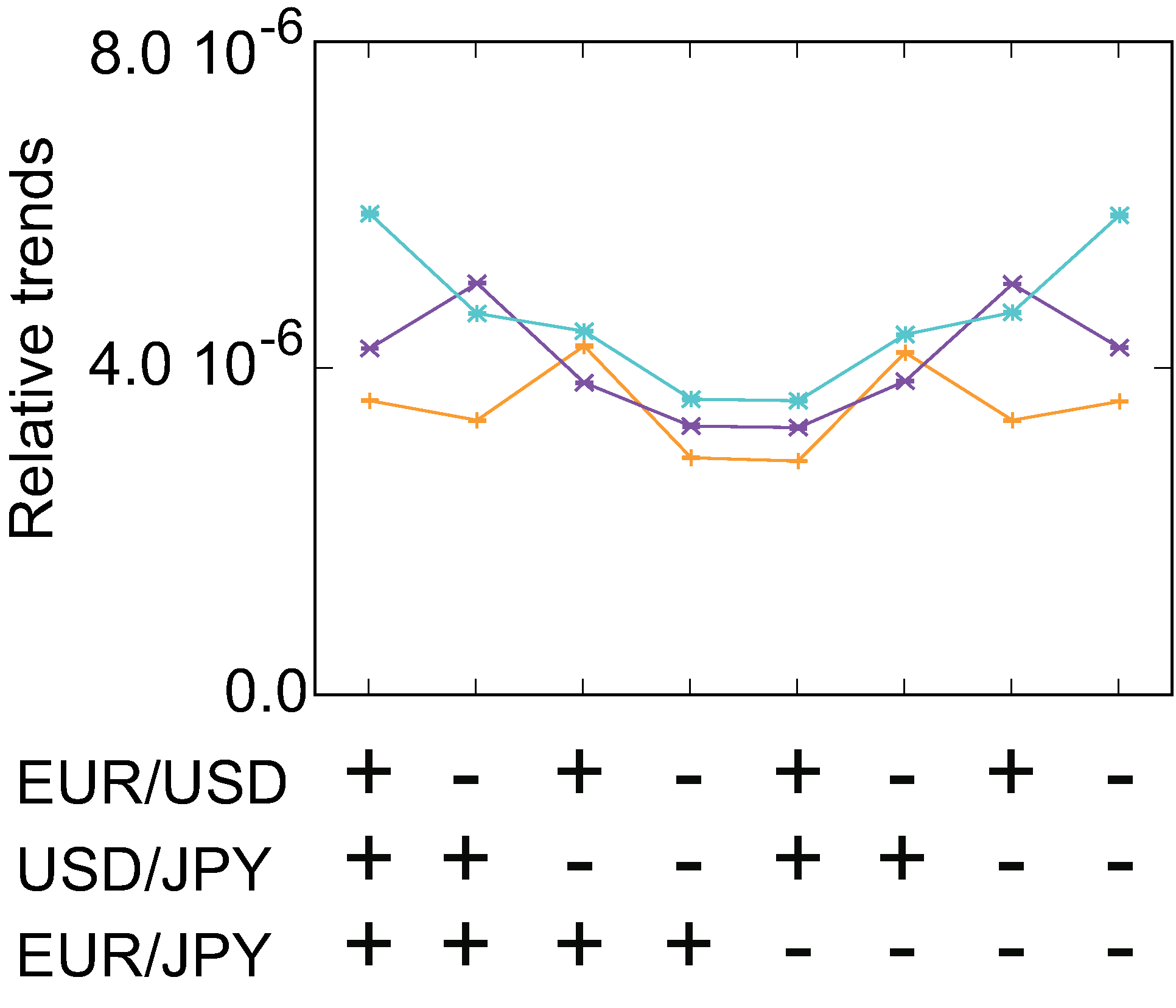

Supplement: S17 Fig — (TIF) [file pone.0234709.s017.tif]
