# Supplementary material for: The microscopic relationships between triangular arbitrage and cross-currency correlations in a simple agent based model of foreign exchange markets
Source: PLoS One. 2020 Jun 24;15(6):e0234709. doi: 10.1371/journal.pone.0234709 (PMC7313750; doi:10.1371/journal.pone.0234709)
Supplement: S1 File — (PDF) [file pone.0234709.s018.pdf]

Supporting information: The microscopic relationships between triangular arbitrage and cross-currency correlations in a simple agent based model of foreign exchange markets

Alberto Ciacci[1,2,¶,*], Takumi Sueshige[3,¶], Hideki Takayasu[4,5], Kim Christensen[1,2], Misako Takayasu[3,4,*]

1 Blackett Laboratory, Imperial College London, London, United Kingdom

2 Center for Complexity Science, Imperial College London, London, United Kingdom

3 Department of Mathematical and Computing Science, School of Computing, Tokyo Institute of Technology, Nagatsuta-cho, Midori-ku, Yokohama, Japan

4 Institute of Innovative Research, Tokyo Institute of Technology, Nagatsuta-cho, Yokohama, Japan

5 Sony Computer Science Laboratories, Higashigotanda, Shinagawa-ku, Tokyo, Japan

¶These authors contributed equally to this work

* Corresponding authors

E-mail: alberto.ciacci16@imperial.ac.uk (AC)
E-mail: takayasu.m.aa@m.titech.ac.jp (MT)

S1 Supporting figures

Fig S1. Mid price patterns. The panels show the dynamics of the mid price between January 1st 2011 and December 31st 2014 in the EUR/USD (top panels), USD/JPY (middle panels) and EUR/JPY (bottom panels) markets. Data is provided by EBS, see Section 2.2.

Fig S2. Dealer Model basics. The market is participated by $N = 4$ market makers providing bid $b_i(t)$ (blue circles) and ask $a_i(t)$ (red circles) quotes, $i = 1, \ldots, N$. The continuous price grid prevents limit orders to be queued at the same price level. Each limit order includes one unit of the traded currency. The market making spread $L = a_i(t) - b_i(t)$, $i = 1, \ldots, N$ is the same for each agent and constant in time, that is, market makers only manage their dealing price $z_i(t) = (a_i(t) + b_i(t))/2$, $i = 1, \ldots, N$ (white squares) to dynamically adjust their bid and ask quotes $b_i(t) = z_i(t) - L/2$ and $a_i(t) = z_i(t) + L/2$, $i = 1, \ldots, N$. The vertical dashed lines mark the best bid $b(t)$ (blue) and ask $a(t)$ (red) quotes. The distance between the best quotes is the current spread $s(t) = a(t) - b(t)$. The current mid price is $m(t) = (a(t) + b(t))/2$.

Fig S3. Interactions in the Dealer Model. (a) Market makers are not engaging in transactions as the best bid price (blue dashed line) is smaller than the best ask price (red dashed line). (b) The best bid price matches the best ask price, prompting Market Makers #1 and #2 to exchange one unit of the traded FX rate (green box). The transaction price is the mid point between the two quotes $p = (a_1 + b_2)/2$. (c) This transaction prompts each market maker to update its dealing price to the latest transaction price (i.e., $z \to p$).

Fig S4. Interactions in the Arbitrager Model. (a) Market makers are not engaging in transactions as the best bid price (blue dashed line) is smaller than the best ask price (red dashed line). (b) The best bid price matches the best ask price, prompting Market Makers #1 and #2 to exchange one unit of the traded FX rate (green box). The transaction price is the mid point between the two quotes $p = (a_1 + b_2)/2$. (c) This transaction prompts the two transacting market makers to re-adjust their dealing prices z to the latest transaction price p.

Fig S5. Exploiting a triangular arbitrage opportunity in the Arbitrager Model. (a) The states of the three markets before the emergence of an exploitable triangular arbitrage opportunity. (b) When $\mu^I(t) \geq 1$, the arbitrager submits a buy market order (blue square) in the EUR/JPY market and sell market orders (red squares) in the EUR/USD and USD/JPY markets, matching the encapsulated limit orders (i.e., Market Maker # 1 in EUR/JPY, Market Maker # 2 in EUR/USD and Market Maker # 3 in USD/JPY). (c) The transacting market makers re-adjust their dealing prices to the quote matched by the arbitrager's market order (e.g., $z_{1,\mathrm{EUR/JPY}}(t + dt) \to a_{1,\mathrm{EUR/JPY}}(t)$), causing a mid price change in each market.

Fig S6. Inverse function sampling in the context of the Arbitrager Model. (a) The LOB profile is stable if the PDF of r corresponds to the triangular function $\psi_\ell(r)$ [1]. (b) The CDF $\Psi_\ell(r)$. (c) Schematic of the inverse function sampling applied to $\Psi_\ell(r)$.

Fig S7. Waiting times statistics in real trading data. Cumulative density functions (CDFs) of the waiting times between consecutive transactions for EUR/USD (red), USD/JPY (blue) and EUR/JPY (green) in (a) 2011, (b) 2012, (c) 2013 and (d) 2014. Data is provided by EBS, see Section 2.2.

Fig S8. Waiting times statistics in the Arbitrager Model. Probability density functions (PDFs) of the waiting times between consecutive transactions for EUR/USD (red), USD/JPY (blue) and EUR/JPY (green) in the Arbitrager Model. Simulations are performed under the same settings of the experiment presented in Fig 5(b), bottom panel. For an adequate comparison against the theoretical predictions of the average time between consecutive transactions [1], the PDFs do not account for transactions triggered by the arbitrager.

Fig S9. Trading data vs. model based cross-correlations functions. Enlarged visualization of the cross-correlation functions $\rho_{i,j}(\omega)$ presented in Fig 5. (a) Real market data (EBS) in 2013. (b) Arbitrager Model simulations. Contrarily to the cross-correlation functions displayed in (a), the model-based $\rho_{i,j}(\omega)$ takes non-zero values when $\omega \to 0$ sec and stabilizes on longer time-scales. Simulations are performed under the same settings of the experiment presented in Fig 5(b), bottom panel. Details on the initialization of the model and the conversion between simulation time (i.e., time steps) and real time (i.e., sec) are provided in S3.2 Section.

Fig S10. Foreseeing a triangular arbitrage opportunity in the Arbitrager Model. The plot considers the EUR/JPY market. The best bid and ask quotes are marked by the blue and red dashed lines, respectively. The implied best bid price of EUR/JPY (i.e., $b_{\mathrm{USD/JPY}} \times b_{\mathrm{EUR/USD}}$) is denoted by the green solid line. (a) The current best ask quote $a_{\mathrm{EUR/JPY}}$ (red dashed line) is smaller than the implied best bid quote $b_{\mathrm{USD/JPY}} \times b_{\mathrm{EUR/USD}}$ (green solid line). This misprice exposes Market Maker #1 to the risk of transacting with the arbitrage who wants to *buy low* $a_{\mathrm{EUR/JPY}}$ and *sell high* $b_{\mathrm{USD/JPY}} \times b_{\mathrm{EUR/USD}}$. (b) When $\chi^{I}_{1,\mathrm{EUR/JPY}} \geq 1 + \zeta_{\mathrm{MM,\ EUR/JPY}}$, Market Maker #1 adjusts its dealing price $z_{1,\mathrm{EUR/JPY}}$ to the mid price $m_{\mathrm{EUR/JPY}}$ (i.e., the mid point between the best quotes in (a)). This action neutralizes the existing triangular arbitrage opportunity as the new best ask quote $a_{\mathrm{EUR/JPY}}$ (red dashed line) matches or exceeds the implied best bid quote $b_{\mathrm{USD/JPY}} \times b_{\mathrm{EUR/USD}}$ (green solid line).

Fig S11. Trading data vs. model based cross-correlation functions. Cross-correlation function $\rho_{i,j}(\omega)$ for ΔUSD/JPY vs. ΔEUR/USD (green), ΔEUR/USD vs. ΔEUR/JPY (blue) and ΔUSD/JPY vs. ΔEUR/JPY (red) as a function of the time-scale ω of the underlying time series. (a) Real market data (EBS) across four distinct years (2011-2014). (b) Extended Arbitrager Model simulations. The number of participating market makers ($N_{\mathrm{EUR/USD}}, N_{\mathrm{USD/JPY}}, N_{\mathrm{EUR/JPY}}$) are $(30, 33, 20)$ in the first experiment, see (b) top panel, and $(30, 27, 20)$ in the second experiment, see (b) bottom panel. Details on the other settings of the simulations are provided in Fig 5. The risk profile of the arbitrager is $\lambda_{\mathrm{A}} = 0.01$ while the risk profiles of market makers are $\lambda_{\mathrm{MM,\ USD/JPY}} = \lambda_{\mathrm{MM,\ EUR/USD}} = \lambda_{\mathrm{MM,\ EUR/JPY}} = 0.001$. The pegging probability in the EUR/JPY market is $\gamma = 0.01$. The insets in (b) provide an enlarged visualization of the cross-correlations functions $\rho_{i,j}(\omega)$ on very short time-scales (i.e. $\omega < 5$ sec). Details on the initialization of the model and the conversion between simulation time (i.e., time steps) and real time (i.e., sec) are provided in S3.2 Section.

Fig S12. Price trend signs and market states. Simulated price patterns of EUR/USD (top), USD/JPY (mid) and EUR/JPY (bottom). Periods of negative (positive) price trends are denoted by a red (green) background. Vertical dashed lines mark a change in the ecology configuration $q(t)$. Price trends $\phi_{n,\ell}$ are calculated over the most recent $n = 15$ changes in the transaction price and with scaling constant $\xi = 5$. The table below the panels combines the market states to show how the ecology configuration $q(t)$ evolves in time.

Fig S13. Complementary cumulative distribution function (CCDF) of the time between the emergence of the first triangular arbitrage opportunity and the transition to another configuration. The CCDFs are presented in two separate panels and each color represents a given configuration: (a) $\{+,+,+\}$ (violet), $\{-,+,+\}$ (cyan), $\{+,-,+\}$ (green) and $\{-,-,+\}$ (orange). (b) $\{-,-,-\}$ (violet), $\{+,-,-\}$ (cyan), $\{-,+,-\}$ (green) and $\{+,+,-\}$ (orange). The black lines mark the CCDF of the interval (in sec) between a random point in time and the transition to another configuration. The y-axis is visualized in the logarithmic scale. Configurations exhibit different tails of the distribution, suggesting that the probability of observing large waiting times between the emergence of the first triangular arbitrage opportunity and the transition to another configuration depends on the current combinations of market states.

Fig S14. Complementary cumulative distribution function (CCDF) of the time required for the first triangular arbitrage opportunity to emerge. The CCDFs are presented in two separate panels and each color represents a given configuration: (a) $\{+,+,+\}$ (violet), $\{+,+,+\}$ (cyan), $\{+,-,+\}$ (green) and $\{-,-,+\}$ (orange). (b) $\{-,-,-\}$ (violet), $\{+,-,-\}$ (cyan), $\{-,+,-\}$ (green) and $\{+,+,-\}$ (orange). The y-axis is visualized in the logarithmic scale. Configurations exhibit different tails of the distribution, suggesting that the probability of observing large waiting times between the inception of the configuration and the emergence of the first triangular arbitrage opportunity depends on the current combinations of market states.

Fig S15. Fraction of triangular arbitrage opportunities of the first and second type in each ecology configuration. Black bars denote the incidence of type 1 opportunities, see Eq. (4a), while white bars represent the incidence of type 2 opportunities, see Eq. (4b). These statistics suggest that one type appears more frequently than the other, depending on the considered configuration.

Fig S16. The sequence of transitions between configurations exhibits a clustered behavior. The x-axis represents an arbitrary time window of the experiment. The y-axis splits the eight ecology configurations in three groups - from top to bottom: (1) cluster 1, (2) cluster 2 and (3) $\{-,-,+\}$ and $\{+,+,-\}$, which are the two configurations that do not belong to any cluster. Details on the concept of configuration clustering are provided in Section 3.2. At each point in time the current ecology configuration is identified and a marker is added to the group it belongs to. A visual inspection of the figure reveals the presence of time windows in which the system moves between configurations belonging to the same cluster, corresponding to the long, uninterrupted lines observed in groups 1 and 2, but not in group 3. These peculiar dynamics favor the appearance of configurations belonging to groups 1 and 2 at the expenses of those belonging to group 3, see Fig 7(b). Details on the conversion between simulation time (i.e., time steps) and real time (i.e., sec) are provided in S3.2 Section.

Fig S17. Price trends and changes in market states. Sample averages of the normalized absolute value of the price trend $\langle|\phi_{n,\ell}(t)|/p_\ell(t_0)\rangle$ for EUR/USD (orange), USD/JPY (violet) and EUR/JPY (cyan). Normalizing by the initial center of mass $p_\ell(t_0)$ allows to compare the price trends across markets with different price magnitudes. The value $|\phi_{n,\ell}(t)|/p_\ell(t_0)$ is exclusively sampled at the emergence of each triangular arbitrage opportunity and each configuration is considered independently. As arbitrager's market orders alter price trends, the value of $|\phi_{n,\ell}(t)|/p_\ell(t_0)$, where t is the time step when μ^I or μ^{II} exceeds the unit, provides an intuitive measure of how currently hard is to flip the state of the ℓ-th market. For instance, consider $\{+,+,+\}$ and observe that $\langle|\phi_{n,\ell}(t)|/p_\ell(t_0)\rangle$ is much higher in EUR/JPY than in EUR/USD and USD/JPY. This is reflected in the probabilities of transitioning from $\{+,+,+\}$ to other configurations. Flipping EUR/JPY before the other two markets, causing a transition to $\{+,+,-\}$, occurs in 22.7% of the cases. However, flipping EUR/USD or USD/JPY first, causing a transition to $\{-,+,+\}$ or $\{+,-,+\}$, occur in 35.8% and 33.6% of the cases respectively, see S5 Table.

S2 Supporting tables

Table S1. Tick sizes adopted in the EBS market.

Initial Month	USD/JPY	EUR/USD	EUR/JPY
2011-03	0.01	0.0001	0.01
2012-09	0.001	0.00001	0.001
-	0.005	0.00005	0.005

EBS has changed the tick size twice in the period between January 1st 2011 and December 31st 2014. The table reports the initial month (yyyy-mm) of each implementation period (first column) and the corresponding tick size in the USD/JPY (second column), EUR/USD (third column) and EUR/JPY (fourth column) markets. See [2] for further details.

Table S2. Parameters governing the dynamics of the Arbitrager Model

**Name**	**Symbol**	**Dimension**	**Section**
Initial center of mass	$p_\ell(t_0)$	price	S3.2.2
Market making spread	L_ℓ	price	S3.2.2
Number of market participants	N_ℓ	dimensionless	S3.2.3
Volatility of dealing price updates	σ_ℓ	price/$\sqrt{\text{time}}$	S3.2.3
Average time between transactions	Γ	time (sec)	S3.2.3
Discretized time step	Δt	time (sec)	S3.2.3
Price changes accounted in $\phi_{n,\ell}(t)$	n	dimensionless	S3.2.4
Scaling of the weight function in $\phi_{n,\ell}(t)$	ξ	dimensionless	S3.2.4
Trend-following strength	c_ℓ	price/time	S3.2.5

The evolution of the Arbitrager Model ecology is controlled by 9 parameters. For each parameter, the table reports its nomenclature, symbol, dimension and the section which provides details on how its value is set in the simulations presented in Fig 5.

Table S3. Initial center of mass and market making spread in each market

**Exchange Rate**	$\boldsymbol{p(t_0)}$	$\boldsymbol{L}$
EUR/USD	1.25	0.05
USD/JPY	110	0.05×(110/1.25)
EUR/JPY	137.5	0.05×(137.5/1.25)

Values of $p_\ell(t_0)$ and L_ℓ for EUR/USD, USD/JPY and EUR/JPY.

Table S4. Approximate equivalences between real and model time

**sec**	**time steps**
1	100
10	1000
60	6000

The results of Kanazawa *et al.* [1,3] are used to establish an approximate equivalence between real and model time. Assuming $\Delta t = 0.01$ sec, the table shows how many time steps roughly equate to 1 sec, 10 sec and 1 min.

Table S5. Transition rates between two configurations

**Configuration**	{+, +, +}	{-, +, +}	{+, -, +}	{-, -, +}	{+, +, -}	{-, +, -}	{+, -, -}	{-, -, -}
{+, +, +}		0.358	0.336	0.010	0.227	0.028	0.026	0.014
{-, +, +}	0.375		0.027	0.218	0.011	0.329	0.014	0.026
{+, -, +}	0.364	0.026		0.217	0.012	0.014	0.341	0.026
{-, -, +}	0.035	0.302	0.295		0.006	0.028	0.030	0.305
{+, +, -}	0.304	0.030	0.028	0.007		0.294	0.302	0.035
{-, +, -}	0.026	0.340	0.014	0.012	0.220		0.026	0.363
{+, -, -}	0.028	0.013	0.330	0.011	0.217	0.027		0.374
{-, -, -}	0.015	0.027	0.027	0.226	0.010	0.335	0.359	

Rows (Columns) indicate the departed (reached) configuration. These transition rates correspond to the total number of transitions between two specific configurations normalized by the total number of transitions from the departed configuration. The two grey portions of the matrix mark the first (upper-left) and second (lower-right) clusters discussed in Section 3.2.

S3 Supporting sections

S3.1 The Dealer Model (Yamada *et al.* 2009)

The Dealer Model [4] introduces a simple market ecology in which N agents interact in a single inter-dealer market where trading is organized in a LOB. For simplicity, the model assumes a continuous price grid, neglecting the role played by the tick size in real financial markets. Agents act as market makers by maintaining buy and sell limit orders through which they provide a bid and an ask quote to the market, see S2 Fig.

Transactions occur when the i-th market maker is willing to buy at a price that matches or exceeds the ask price of the j-th market maker (i.e., $b_i \geq a_j$). Trades are settled at the transaction price $p(g_t) = (a_j(t) + b_i(t))/2$, where g_t is the number of transactions occurred in $[0, t[$. It is important to stress that the mid-price $m(t)$ and the transaction price $p(g_t)$ are two different quantities. The former, being the mid point

between the best quotes, is the *center* of the LOB and can be tracked at any time step. The latter is sampled whenever two market makers engage in a trade.

The Dealer Model assumes that a transaction prompts the entire market to immediately update their dealing prices $z_i(t+dt),\ i=1,\dots,N$ to the latest transaction price $p(g_t)$, see S3 Fig. In the absence of interactions, market makers independently update their dealing prices by adopting a trend-based strategy

$$\frac{dz_i(t)}{dt} = c\langle\Delta p\rangle_n + \sigma\epsilon_i(t), \quad i=1,\dots,N \tag{S1}$$

where $\sigma > 0$ and $\epsilon_i(t)$ is a Gaussian white noise. The term

$$\langle\Delta p\rangle_n = \frac{2}{n(n+1)}\sum_{k=0}^{n-1}(n-k)(p(g_t-k)-p(g_t-k-1)), \tag{S2}$$

is a weighted average of the last $n < g_t$ changes in the transaction price p.

The real-valued parameter c controls how the current price trend $\langle\Delta p\rangle_n$ influences market makers' strategies. For instance, $c > 0$ represents a market maker that tends to adjust its dealing price $z(t)$ in the direction of the price trend (i.e., trend-following). Conversely, $c < 0$ characterizes a market maker that tends to adjust its dealing price in the opposite direction of the price trend (i.e., contrarian).

S3.2 Initialization and dynamic control of the Arbitrager Model

S3.2.1 Introduction

Kanazawa *et al.* [3] have recently introduced a microscopic model of the interactions between high frequency traders (HFTs) and investigated its theoretical aspects by adapting Boltzmann and Langevin equations to this specific context. The results of this work have been further formalized in a parallel study from the same authors [1]. The dealing price updates in the HFT model are driven by the following dynamics

$$\frac{dz_{i,\ell}(t)}{dt} = c_\ell^*\tanh\left(\frac{p_\ell(g_{t,\ell})-p_\ell(g_{t,\ell}-1)}{\Delta p_\ell^*}\right) + \sigma_\ell\epsilon_{i,\ell}(t), \quad i=1,\dots,N_\ell \tag{S3}$$

where Δp_ℓ^*, c_ℓ^* are constants while the other variables and constants have the same meaning as in Eq. (5). It can be shown that setting $\Delta p_\ell^* \gg p_\ell(g_{t\ell}) - p_\ell(g_{t\ell}-1)$ allows for a linear approximation of Eq. (S3) that resembles the dynamics of the dealing price updates in the Arbitrager Model, see Eq. (5). This correspondence allows to exploit the theoretical results of [1,3] to achieve a satisfactory control of the dynamics of the Arbitrager Model. For instance, S8 Fig shows that the average time between consecutive transactions in simulations of the Arbitrager Model is in strong agreement with its theoretical value estimated in the framework of Kanazawa *et al.* [1,3]. The following sections provide details on how the parameters governing the evolution of the Arbitrager Model, see S2 Table, have been set in the simulations discussed in this study.

S3.2.2 Initial state of the LOB

To initialize the ℓ-th LOB, the first step consists in fixing its initial center of mass $p_\ell(t_0)$ and the constant market making spread L_ℓ. The former is set arbitrarily to a value with the same magnitude of the mid-price patterns observed in real trading data, see S1 Fig. Following the analysis of [3], the market making spread in the USD/JPY market is fixed to $L_{\mathrm{USD/JPY}} = 0.05$. For simplicity, the market making spread in other markets is set such that it becomes proportional to the size of $p_\ell(t_0)$, that is $L_\ell = L_{\mathrm{USD/JPY}} \times (p_\ell(t_0)/p_{\mathrm{USD/JPY}}(t_0))$.

At this point the values in S3 Table are used to obtain the initial dealing prices for each trader and market, thus revealing the initial profile of the LOBs

$$z_{i,\ell}(t_0) = \begin{cases} \frac{L_\ell}{2}\left(\sqrt{2u_{i,\ell}} - 1\right) + p_\ell(t_0), & \text{if } 0 < u_{i,\ell} \leq 0.5. \\ \frac{L_\ell}{2}\left(1 - \sqrt{2(1-u_{i,\ell})}\right) + p_\ell(t_0), & \text{otherwise}, \end{cases} \tag{S4}$$

where $u_{i,\ell} \sim U(0,1)$ is an uniformly distributed random variable. The expression in Eq. (S4) is derived from the inverse function sampling procedure, see S6 Fig. Let $r \equiv (z(t_0) - p(t_0))$ be the relative distance between an initial dealing price $z(t_0)$ and the initial center of mass price $p(t_0)$. The LOB profile is stable when the probability density function (PDF) of r is

$$\psi_\ell(r) = \begin{cases} \frac{2}{L_\ell}\left(1 - |\frac{2r}{L_\ell}|\right), & \text{if } |r| \leq \frac{L_\ell}{2}. \\ 0, & \text{otherwise}. \end{cases} \tag{S5}$$

It follows that the cumulative density function (CDF) of r is

$$\Psi_\ell(r) = \begin{cases} \frac{1}{2L_\ell^2}\left(L_\ell + 2r\right)^2, & \text{if } -\frac{L_\ell}{2} < r \leq 0. \\ -\frac{1}{2L_\ell^2}\left(L_\ell - 2r\right)^2 + 1, & \text{otherwise}. \end{cases} \tag{S6}$$

Then, the inverse function of Eq. (S6) is computed

$$\Psi_\ell^{-1}(y) = \begin{cases} \frac{L_\ell}{2}\left(\sqrt{2y} - 1\right), & \text{if } 0 < y \leq 0.5. \\ \frac{L_\ell}{2}\left(1 - \sqrt{2(1-y)}\right), & \text{if } 0.5 < y \leq 1. \end{cases} \tag{S7}$$

Finally, assuming that $y = u \sim U(0,1)$, Eq. (S7) is used to obtain the value of the initial dealing price $z_{i,\ell}(t_0)$, see Eq. (S4).

S3.2.3 Relationships between simulation time and real time

Kanazawa *et al.* [1] found that the average time between two consecutive transactions is

$$\Gamma = \frac{L_\ell^2}{2N_\ell\sigma_\ell^2}. \tag{S8}$$

For the sake of simplicity, the Arbitrager Model relies on the assumption that the three markets *moves at the same pace*, on average. This implies that Γ is the same in each market and constant in time. The parameter Γ is derived from real trading data. First, the average waiting times between consecutive transactions are calculated in each market and for each trading year. This leads to 12 averages (i.e., 4 years $\times$ 3 FX rates). Finally, the median of these averages is computed to obtain a common value for Γ. In the dataset employed in this study it turns out that $\Gamma \approx 0.7$ sec, see S7 Fig. To ensure that simulations of our model maintain $\Gamma \approx 0.7$ sec, the remaining free parameters N_ℓ and σ_ℓ must be fixed such that Eq. (S8) is satisfied. The number of market makers participating each market is set heuristically by considering several combinations ($N_{\mathrm{EUR/USD}}$,$N_{\mathrm{USD/JPY}}$,$N_{\mathrm{EUR/JPY}}$) and examining how well the model-based cross-correlation function $\rho_{i,j}(\omega)$ replicates the same function built on real trading data. Having fixed N_ℓ, the volatility of the dealing price updates

$$\sigma_\ell = \frac{L_\ell}{\sqrt{2N_\ell\Gamma}} \tag{S9}$$

is found by rearranging Eq. (S8). Finally, the amplitude of a discretized time step in the model simulation Δt should be set such that $\Delta t \ll \Gamma$. Therefore, this parameter is fixed

to $\Delta t = 0.01$ sec and used in the discrete approximation of Eq. (5). S8 Fig shows the distributions of the time between consecutive transactions in simulations of the Arbitrager Model. The average waiting time is $\tilde{\Gamma} \approx 0.65$ sec (65 time steps), which is very close to the theoretical value $\Gamma \approx 0.7$ sec. Acknowledging the simplifications that characterize the Arbitrager Model (e.g., Γ is the same in each market), the approximate equivalence $\Delta t = 0.01$ sec is used to convert simulation time steps in real time, see S4 Table, and compare the stabilization of the data-based and model-based cross-correlation functions $\rho_{i,j}(\omega)$, see Fig 5 and S11 Fig.

S3.2.4 Parameters involved in the calculation of the current price trend

The calculation of the price trend process $\phi_{n,\ell}(t)$, see Eq. (6), relies on two parameters: the number of accounted transaction price changes n and the scaling constant of the exponential weighting function ξ. In the simulations presented in Fig 5 and S11 Fig these are arbitrarily set to $n = 15$ observations and $\xi = 5$. These choices allow to model a scenario in which trend-following market makers do not exclusively rely on the latest change in the transaction price to determine the current *direction* of the market. Instead, they compute a weighted average of the most recent price changes where weights are calculated according to an exponential function.

S3.2.5 Trend-following strength parameter

The trend-following strength parameter c determines how the sign and value of the current price trend $\phi_{n,\ell}(t)$ affect the strategic decisions of the participating market makers. When $c > 0$, market makers are likely to update their dealing prices $z(t)$ upward when the price trend is positive and downward when the price trend is negative. Conversely, $c < 0$ indicates that market makers are more likely to update their dealing prices in the opposite direction of the price trend sign.

Recently, Sueshige *et al.* [5] have classified the strategic behavior of FX traders by examining EBS data covering the trading activity in the USD/JPY market during the week starting from June 5th 2016. They found that a significant fraction of traders adopt trend-following strategies (i.e., $c > 0$). This observation is consistent with the model of Yura *et al.* [6].

Relying on these studies, the assumption that market makers populating the Arbitrager Model ecology adopt trend-following strategies (i.e., $c > 0$) is enforced. For simplicity, c is the same for every market maker and across markets. This parameter is fixed according to Eq. (91) in [1]

$$\Delta\tilde{p}^* = 1/(c\Gamma), \tag{S10}$$

where $\Delta\tilde{p}^*$ is a non-dimensional parameter. Furthermore, $\Delta\tilde{p}^*$ shall take values that are not far from 2 for the model to produce the marginal trend-following behavior, which successfully replicated various statistical properties of real trading data in [1]. This allows to set $c = 0.8$, thus obtaining $\Delta\tilde{p}^* \approx 1.79$.

S3.3 An extended version of the Arbitrager Model

S3.3.1 Motivations

The Arbitrager Model qualitatively replicates the shape of the cross-correlation functions $\rho_{i,j}(\omega)$ and provides important insights on how the microscopic interactions between market makers and arbitragers entangles the dynamics of different FX rates. However, the cross-correlation functions $\rho_{i,j}(\omega)$ reproduced by this extremely simple model present two features that are not found in real trading data. First, on extremely

short time-scales (i.e., $\omega \to 0$ sec) the model-based $\rho_{i,j}(\omega)$ does not approach zero as the same function built on real trading data. Second, the model-based $\rho_{i,j}(\omega)$ flattens when $\omega \gtrapprox 30$ sec while the data-based $\rho_{i,j}(\omega)$ flattens when $\omega \gtrapprox 10$ sec, see S9 Fig.

It is plausible that these differences stem from the extreme simplicity of the Arbitrager Model. To verify this assertion, a modified version of the model which mimics more features of real FX markets is introduced in this section. This extended, more realistic framework retains the same fundamental rules of the Arbitrager Model, that is, i) market makers continuously provide liquidity in a single market and ii) the arbitrager is the only agent allowed to operate across markets through the submission of predatory market orders. However, it also adds three distinct features inspired by real markets practices. First, agents' responses to triangular arbitrage opportunities emerge from a more rational decision making process in which they take into the account the risks associated to the implementation of this strategy. Second, market makers foresee predatory market orders and re-adjust their quotes in advance, reducing the likelihood of being matched by arbitragers' orders. Third, market makers operating in the EUR/JPY market peg their quotes to the implied best bid and ask prices with probability γ. This introduces an additional toy (i.e., unrealistic) mechanism through which the dynamics of different FX rates become entangled.

S3.3.2 The arbitrager

In the original model, see Section 2.3, the arbitrager automatically submits predatory market orders as soon as Eqs. (4a) or (4b) exceeds the unit. In real FX markets this decision is far less trivial as these orders might not be executed at the prices used in the calculation of Eqs. (4a) and (4b). For instance, faster traders could have already exploited the existing opportunity, pushing back Eqs. (4a) or (4b) below the unit. As a result, the profitable misprice evaporates, exposing slower arbitragers to the risk of generating losses. To address this limitation, the extended Arbitrager Model introduces a more realistic decision making process in which the arbitrager takes into the account the risks associated with this trading strategy. In particular, the arbitrager submits market orders if one of the following conditions is satisfied

$$\mu^{I}(t) \geq 1 + \zeta_{\mathrm{A}}(t), \tag{S11a}$$

$$\mu^{II}(t) \geq 1 + \zeta_{\mathrm{A}}(t), \tag{S11b}$$

where $\zeta_{\mathrm{A}}(t) \sim \exp(\lambda_{\mathrm{A}})$. The parameter λ_{A} represents the risk profile of the arbitrager. The higher the value of λ_{A}, the more profitable the gap between real and implied prices must be to *convince* the arbitrager to exploit the current opportunity.

S3.3.3 Market makers

The submission of predatory market orders ensures immediate execution, forcing the matched market makers to either sell *too low* or buy *too high.* In the original Arbitrager Model, see Section 2.3, market makers remain indifferent to triangular arbitrage opportunities, that is, they do not attempt to anticipate the arbitrager to avoid predatory market orders. However, it is plausible that such a simplifying assumption does not adequately describe the behavior of liquidity providers acting in real FX markets. In this extension of the Arbitrager Model the strategic behaviors of market makers are enhanced by allowing them to foresee the arbitrager's moves and re-adjust their quotes accordingly. Their dealing price updates are driven by Eq. (5), however, they also track the likelihood of engaging in an unfavourable transaction with the arbitrager. For instance, the i-th market maker operating in the EUR/JPY market

monitors its exposure to predatory market orders by calculating the following ratios

$$\chi^{I}_{i,\mathrm{EUR/JPY}}(t) = \frac{b_{\mathrm{USD/JPY}}(t) \times b_{\mathrm{EUR/USD}}(t)}{a_{i,\mathrm{EUR/JPY}}(t)}, \tag{S12a}$$

$$\chi^{II}_{i,\mathrm{EUR/JPY}}(t) = \frac{b_{i,\mathrm{EUR/JPY}}(t)}{a_{\mathrm{USD/JPY}}(t) \times a_{\mathrm{EUR/USD}}(t)}, \tag{S12b}$$

where $b_{i,\ell}(t)$ and $a_{i,\ell}(t)$ are the current bid and ask limit prices of the i-th market maker and $b_\ell(t)$ and $a_\ell(t)$ are the current best quotes in the ℓ-th market. Clearly, Eqs. (S12a) and (S12b) can be straightforwardly rewritten for market makers operating in the USD/JPY or EUR/USD markets.

The more Eqs. (S12a) or (S12b) exceeds the unit, the larger the discrepancy between the current quote of the i-th market maker and the implied best cross FX rate. In the former case, the i-th market maker is underpricing EUR/JPY, facing the risk of selling *too low*. In the latter case, the i-th market maker is overpricing EUR/JPY, facing the risk of buying *too high*. As the implied cross FX rate is the same for every agent, the market maker with the highest value of χ is always the one who is offering the best quote, hence the first to be matched by predatory market orders.

In the same spirit of Eqs. (S11a) and (S11b), the i-th market maker, perceiving a high risk of interacting with the arbitrager, deletes and re-adjusts its current quotes if one of the following conditions is satisfied

$$\chi^{I}_{i,\mathrm{EUR/JPY}}(t) \geq 1 + \zeta_{\mathrm{MM,\ EUR/JPY}}(t), \tag{S13a}$$

$$\chi^{II}_{i,\mathrm{EUR/JPY}}(t) \geq 1 + \zeta_{\mathrm{MM,\ EUR/JPY}}(t), \tag{S13b}$$

where $\zeta_{\mathrm{MM,\ EUR/JPY}}(t) \sim \exp(\lambda_{\mathrm{MM,\ EUR/JPY}})$. The parameter $\lambda_{\mathrm{MM,EUR/JPY}}$ represents the average risk profile (i.e., is the same for every market maker) in the EUR/JPY market. The lower the value of $\lambda_{\mathrm{MM,EUR/JPY}}$, the less market makers tolerate their exposure to predatory market orders.

When Eqs. (S13a) or (S13b) is satisfied, the i-th market maker sets its dealing price to the current mid price $m_{\mathrm{EUR/JPY}}(t) = (a_{\mathrm{EUR/JPY}}(t) + b_{\mathrm{EUR/JPY}}(t))/2$, rejecting the update imposed by Eq. (5) to reduce the risk of engaging in a transaction with the arbitrager, see S10 Fig. This mimics real traders deleting their limit orders queued in the very first levels of the LOB to replace them with new orders lying farther away from the current best quotes.

S3.3.4 An additional price-entangling mechanism

The law of one price states that in frictionless markets the prices of two assets with the same cash flows must be identical [7]. The law of one price is maintained by two distinct mechanisms, triangular arbitrage and *shopping around*, which promptly correct temporary gaps between the prices of two identical assets [7]. The former has been extensively described in Section 2.1.2 and it is the only way to enforce the law of one price in the standard version of the Arbitrager Model, see Section 2.3. The latter mechanism relates to the fact that rational traders, having detected two assets with identical cash flows but different prices, always buy the one with lower price and sell the one with higher price. This alters the demand and supply in the markets in which these assets are exchanged, thus closing the gap between their prices [7]. Reproducing the shopping around mechanism in the Arbitrager Model requires market makers to operate in multiple LOBs. To avoid a complete overhaul of the fundamentals of the Arbitrager Model, its extension includes a simpler stylized mechanism which retains the basic feature that distinguishes shopping around from triangular arbitrage, that is, the

absence of a *round trip* (e.g. JPY → EUR → USD → JPY) [7]. Market makers operating in the EUR/JPY market peg their bid and ask quotes to the implied best bid and ask prices with constant probability γ, thus rejecting the dealing price update imposed by Eq. (5). For instance, the quotes of the i-th market maker that decides to peg its prices to the implied best quotes at time t are

$$b_{i,\mathrm{EUR/JPY}}(t) = b_{\mathrm{EUR/USD}}(t) \times b_{\mathrm{USD/JPY}}(t), \quad \text{(S14)}$$

$$a_{i,\mathrm{EUR/JPY}}(t) = a_{\mathrm{EUR/USD}}(t) \times a_{\mathrm{USD/JPY}}(t). \quad \text{(S15)}$$

This introduces an additional, simplistic mechanism through which the price of EUR/JPY is pushed towards its implied FX cross rate EUR/USD×USD/JPY.

S3.3.5 Cross-correlation functions and discussion

The inclusion of additional features of real FX markets improves the replication of the characteristic shape of $\rho_{i,j}(\omega)$. In particular, both the data-based and model-based cross-correlation functions $\rho_{i,j}(\omega)$ approach zero on extremely short time-scales (i.e., $\omega \to 0$ sec), see insets of S11(b) Fig. Furthermore, the model-based $\rho_{i,j}(\omega)$ flattens on much shorter time-scales when compared to the standard Arbitrager Model, see Fig 5(b) vs. S11(b) Fig. This rapid stabilization is indeed observed in cross-correlation functions derived from real trading data, see S11(a) Fig.

These results suggest that the discrepancies between model-based and data-based cross-correlation functions stem from the extreme simplicity of the Arbitrager Model which neglects several features and practices of real FX markets. Nonetheless, the standard Arbitrager Model succeeds in providing a comprehensive and intriguing explanation on how the dynamics of different FX rates are entangled at a microscopic level. This result is remarkable, considering the limited number of input parameters and straightforward settings that characterize this model.

The effort of extending the Arbitrager Model provides few important insights. First, the inclusion of reacting market makers corrects the behavior of $\rho_{i,j}(\omega)$ when $\omega \to 0$ sec, that is, the model-based cross-correlation function collapses to zero as in real trading data. The key difference between arbitragers and market makers reactions to triangular arbitrage opportunities is that the former prompt simultaneous transactions, causing mid price changes in each market, while the latter cause a sequence of asynchronous mid price changes and eventually transactions. The characteristic shape of $\rho_{i,j}(\omega)$ presented in S11(b) Fig is based on a set of risk profile parameters that gives market makers a predominant role at the expense of the arbitrager. This means that a large fraction of triangular arbitrage opportunities are neutralized by market makers before the arbitrager can place predatory market orders. This result should not be interpreted as an estimate of the fraction of opportunities that are destroyed by market makers or exploited by arbitragers in real FX markets. However, it suggests that the entanglement of the dynamics of FX rates starts at different times in each market, depending on the current state of the LOB.

The second insight emerging from S11 Fig is that the interdependencies among currencies stem from the interplay of several agents' behaviors. While the interactions between triangular arbitrage and trend-following strategies retain a primary, necessary role in the entanglement of FX rates dynamics, the introduction of a second, complementary mechanism (i.e., *shopping around*) to close the gap between real and implied prices allows the model based $\rho_{i,j}(\omega)$ to stabilize on shorter time-scales ω, obtaining a characteristic shape that is strongly compatible with the same function derived from real trading data. This suggests that in real FX markets additional strategies are likely to interact with triangular arbitrage and trend-following behaviors to shape the features of cross-currency correlations.

References

1. Kanazawa K, Sueshige T, Takayasu H, Takayasu M. Kinetic theory for financial Brownian motion from microscopic dynamics. Physical Review E. 2018;98(5):052317.

2. Mahmoodzadeh S, Gençay R. Tick size change in the wholesale foreign exchange market; 2014.

3. Kanazawa K, Sueshige T, Takayasu H, Takayasu M. Derivation of the Boltzmann equation for financial Brownian motion: Direct observation of the collective motion of high-frequency traders. Physical Review Letters. 2018;120(13):138301.

4. Yamada K, Takayasu H, Ito T, Takayasu M. Solvable stochastic dealer models for financial markets. Physical Review E. 2009;79(5):051120.

5. Sueshige T, Kanazawa K, Takayasu H, Takayasu M. Ecology of trading strategies in a forex market for limit and market orders. PloS one. 2018;13(12):e0208332.

6. Yura Y, Takayasu H, Sornette D, Takayasu M. Financial brownian particle in the layered order-book fluid and fluctuation-dissipation relations. Physical Review Letters. 2014;112(9):098703.

7. Sercu P. International finance: Theory into practice. Princeton University Press; 2009.
